# Circular RNA circHERC4 as a novel oncogenic driver to promote tumor metastasis via the miR-556-5p/CTBP2/E-cadherin axis in colorectal cancer

**DOI:** 10.1186/s13045-021-01210-2

**Published:** 2021-11-15

**Authors:** Jiehua He, Ziqiang Chu, Wei Lai, Qiusheng Lan, Yujie Zeng, Daning Lu, Shaowen Jin, Heyang Xu, Pengwei Su, Dong Yin, Zhonghua Chu, Lu Liu

**Affiliations:** 1 Guangdong Provincial Key Laboratory of Malignant Tumor Epigenetics and Gene Regulation, Guangdong-Hong Kong Joint Laboratory for RNA Medicine, Sun Yat-Sen Memorial Hospital, Sun Yat-Sen University, Guangzhou, 510120 People’s Republic of China; 2 Medical Research Center, Sun Yat-Sen Memorial Hospital, Sun Yat-Sen University, 107 Yan-Jiang Xi Road, Guangzhou, 510120 People’s Republic of China; 3 Department of Gastrointestinal Surgery, Sun Yat-Sen Memorial Hospital, Sun Yat-Sen University, 107 Yan-Jiang Xi Road, Guangzhou, 510120 Guangdong People’s Republic of China

**Keywords:** Colorectal cancer, circHERC4, Metastasis, miR-556-5p, CTBP2, E-cadherin

## Abstract

**Background:**

The main cause of death in colorectal cancer patients is metastasis. Accumulating evidences suggest that circRNA plays pivotal roles in cancer initiation and development. However, the underlying molecular mechanisms of circRNAs that orchestrate cancer metastasis remain vague and need further clarification.

**Methods:**

Two paired CRC and adjacent normal tissues were used to screen the upregulated circRNAs by circRNA-seq; then, cell invasion assay was applied to confirm the functional invasion-related circRNAs. According to the above methods, circHERC4 (hsa_circ_0007113) was selected for further research. Next, we investigated the clinical significance of circHERC4 in a large cohort of patients with CRC. The oncogenic activity of circHERC4 was investigated in both CRC cell lines and animal xenograft studies. Finally, we explored the molecular mechanisms underlying circHERC4 as a malignant driver.

**Results:**

We demonstrated that circHERC4 was aberrantly elevated in CRC tissues (*P* < 0.001), and was positively associated with lymph node metastasis and advanced tumor grade (*P* < 0.01). Notably, the expression of circHERC4 was associated with worse survival in patients with CRC. Silencing of circHERC4 significantly inhibited the proliferation and migration of two highly aggressive CRC cell lines and reduced liver and lung metastasis in vivo. Mechanistically, we revealed that circHERC4 inactivated the tumor suppressor, miR-556-5p, leading to the activation of CTBP2/E-cadherin pathway which promotes tumor metastasis in CRC.

**Conclusions:**

CircHERC4 exerts critical roles in promoting tumor aggressiveness through miR-556-5p/CTBP2/E-cadherin pathway and is a prognostic biomarker of the disease, suggesting that circHERC4 may serve as an exploitable therapeutic target for patients with CRC.

**Supplementary Information:**

The online version contains supplementary material available at 10.1186/s13045-021-01210-2.

## Background

Colorectal cancer (CRC) is one of the most commonly diagnosed cancers and a leading cause of cancer-related deaths worldwide according to the latest global cancer statistics [1]. Until now, surgical resection is still the primary radical treatment for CRC. Despite great advancements in clinical therapeutic strategies targeting CRC, metastasis still occurs in more than 50% of patients undergoing resection [2, 3]. Therefore, it is crucial to explore and better understand the molecular mechanisms that drive the progression of CRC. Moreover, discovering molecular drivers of CRC progression is helpful to improve clinical management through better prognostic predictions and potentially more effective targeted treatments.

Circular RNAs (circRNAs) are defined as a class of non-coding RNAs that mainly derives from the back-splicing of precursor mRNA (pre-mRNA) transcripts and are characterized by a covalently closed loop structure [4–6]. CircRNAs once were considered inconsequential byproducts of the biological process and thought to have no functions. Recently, with the development of deep RNA sequencing technology [7], large amounts of circRNAs have been discovered, revealing that circRNAs could be coded from exons or introns and have multiple regulatory characters in different biological processes and various diseases [8–10]. Recent researches have shown that circRNAs’ expression varied in different pathological responses, especially cancers, such as proliferation, migration, invasion and apoptosis, and could serve as a potential biomarker [11]. The emerging studies have indicated that circRNAs had multiple microRNA (miRNA)-binding sites and could act as endogenous miRNA sponges, known as the competitive endogenous RNAs (ceRNAs) [12, 13], and microRNAs have been found to act as oncogenes or tumor suppressors in different type of cancer [14]. For instance, circHIPK2 can induce autophagy and endoplasmic reticulum stress to regulate the activation of astrocytes in neuroinflammatory disorders by targeting miR124-2HG [15]. CircPRMT5 was overexpressed in a urothelial carcinoma of the bladder and could promote metastasis by sponging miR-30c to induce epithelial–mesenchymal transition [16]. Moreover, circRNAs can also interact with RNA-binding proteins [17, 18], and they splice the target genes and participate in the translation of protein [9, 19]. Emerging evidence shows that circRNA is involved in the development of tumors [20, 21]. However, the functions and underlying mechanisms of certain dysregulated circRNAs in CRC remain largely unknown and the specific characteristics of circRNAs provide a novel potential biomarker and therapeutic target during tumorigenesis and progressions of cancer [22].

In the present study, we characterized a novel circRNA, circHERC4, that was upregulated in CRC tissues by using a circRNA microarray profiling, suggesting an oncogenic role of tumorigenesis and progression. High expression of circHERC4 was positively associated with poor survival of CRC patients. Furthermore, we revealed that miR-556-5p could be sponged by circHERC4. Further mechanism research suggested that miR-556-5p could act as a tumor suppressor in CRC via post-transcriptional inhibition of its target gene, C-terminal-binding protein 2 (CTBP2). CTBP2 was a transcriptional corepressor that participated in several essential cellular processes. There are strong evidences that CTBP2 could promote cancer cell survival and migration/invasion by targeting epithelial and proapoptotic genes and multiple growth inhibitory tumor suppressors [23–26]. Previous study demonstrated that CTBP2 could target the promoter of E-cadherin via several repressors including zinc-finger E-box binding homeobox (ZEB) and induce the downregulation of E-cadherin expression [27]. In conclusion, our study demonstrated that circHERC4 could act as an oncogenic driver of CRC through a novel circHERC4/miR-556-5p/CTBP2/E-cadherin axis and could be exploited in cancer therapy.

## Methods

### CRC clinical data

CRC tissues and matched adjacent normal tissues were derived from 120 CRC patients who underwent radical resection (no chemotherapy or radiotherapy before surgery) at Sun Yat-Sen Memorial Hospital, Sun Yat-Sen University, from 2017 to 2019. All specimens were snap-frozen in liquid nitrogen after removal from patients and were stored at − 80 °C for further use. A tissue cDNA microarray (TMA) containing 64 CRC tissues (MecDNA-HColA095Su01) was purchased from the Shanghai Outdo Biotech Co, Ltd. (Shanghai, China). Approval for this study was provided by the Ethical Review Committee of Sun Yat-Sen Memorial Hospital, Sun Yat-Sen University. The specimens were defined according to the TNM classification. Written informed consent was obtained from each patient before the study began.

### Sanger sequencing

Sanger sequencing by Forevergen (Guangzhou, China) was applied to confirm the amplification products of circRNAs.

### RNase R treatment

RNase R (Epicentre Technologies, USA) was applied to digest linear RNA. RNAs extracted from DLD-1 and HCT116 cells were divided into two groups: one for RNase R treatment and another for control. The sample was incubated for 30 min in 37 °C with 3U/μg of RNase R. For analysis, qRT-PCR was used to detect the expression of HERC4 and circHERC4. GAPDH in the control group was used as the internal reference. Three independent experiments were applied in triplicate.

### Actinomycin D assay

1 × 10^5^ cells per well of DLD-1 and HCT116 cells were seeded in a 6-well plate overnight and 2 mg/L actinomycin D (Sigma, USA) was added into the well for 4, 8, 12 and 24 h. The cells were harvested according to the time of treatment. Then qPCR was performed to analyze the stability of HERC4 mRNA and circHERC4. Three independent experiments were applied in triplicate.

### Cell culture and transfection

Five CRC cell lines (HCT116, DLD-1, HT29, LoVo, SW480) and normal colonic epithelial cells (HCoEpic) were purchased from Chinese Academy of Sciences, Shanghai Institutes for Biological Sciences, and maintained in RPMI-1640 medium (Gibco, Shanghai, China) with 10% FBS (GIBCO, Brazil) at 37 °C with 5% CO_2_. Small interfering RNAs (circHERC4 siRNA, CTBP2 siRNA), miR-556-5p mimics, as well as their corresponding control oligonucleotides were purchased from GenePharma (GenePharma Corporation, Shanghai, China). Transfection was carried out using reagent Lipofectamine RNAimax (Thermo Fisher Scientific Inc. Massachusetts, USA) based on the manufacturer's instructions. SiRNA sequence is available in Additional file 1: Table S3.

### RNA isolation and qRT-PCR

Total RNA from whole-cell lysates was isolated using the TRIzol (TaKaRa, Japan) according to the manufacturer’s protocol. Reverse transcription was performed by using the PrimeScript RT Reagent Kit (Takara, China). Bulge-loop™ miRNA RT-qPCR Primers were applied to determine the level of miRNAs. Quantitative reverse transcription PCR (qRT-PCR) were performed on an ABIPRISMVR 7300 Sequence Detection System (Applied Biosystems). The specific primers are listed in Additional file 1: Tables S1 and S2.

### Western blot analysis

Western blotting was performed as previously reported [28]. The primary antibodies anti-CTBP2 (13,256, Rabbit polyclonal antibody, CST, Danvers, MA, USA), anti-E-cadherin (20,874, Rabbit polyclonal antibody, CST, Danvers, MA, USA) and anti-GAPDH (5174, Rabbit polyclonal antibody, CST, Danvers, MA, USA) were used. The membranes were incubated with goat anti-rabbit secondary antibody (CST) and visualized using chemiluminescent reaction kit (Thermo, USA).

### Cell counting kit-8 proliferation assay

CRC cells’ proliferation ability was measured by CCK8 assay (APExBIO, Houston, USA). Cells (1000 cells per well) in the logarithmic growth phase were seeded into 96-well plates, and 10 μL of cell counting kit-8 (CCK8) solution was added into each well, followed by an incubation at 37 °C for 2 h. The absorbance at the wavelength of 450 nm was spectrophotometrically calculated every 48 h for 3 times.

### Colony formation assay

CRC cells were collected and 1 × 10^3^ cells were seeded into 6-well plates and incubated at 37 °C for 10 days. After the removal of the culture medium, cells were fixed with methanol and stained with 0.1% crystal violet. The number of colonies were then counted and recorded.

### Cell migration and invasion assay

For cell migration assays, cell suspensions (1 × 10^5^ cells) were diluted in 0.2 mL of serum-free RPMI-1640 medium and added to the upper transwell chamber and incubated for 20 h. Cell invasion assay was performed using the transwell chamber coated with the Matrigel (BD Bioscience). The transfected cells were routinely incubated for 48 h. After the incubation, cells were fixed with formaldehyde for 30 min and stained with crystal violet. After washing with PBS, the cells threaded through the filter membrane were counted through a microscope. Five fields were randomly selected for cell counting, and the migration and invasion rates were quantified.

### Luciferase reporter assay

We used a dual-luciferase reporter assay to confirm the direct binding between circHERC4 and miRNAs. PsiCHECK2 (IGE Biotech, China) vector included firefly luciferase gene (hLuc+) and renilla luciferase gene (hRluc). The sequence of circHERC4 was cloned into the psiCHECK2 vector. NC vector or circHERC4 vector was co-transfected with each miRNA mimics. The relative values of hLuc+ and hRluc were measured by Centro LB960 XS3 (Berthold, German). Luciferase reporter assay was applied to explore whether CTBP2 was the direct target of miR-556-5p. The 3’UTR sequence of CTBP2 was cloned into the pcDNA3.0 vector. Subsequently, CTBP2 vector was co-transfected with NC or miR-556-5p mimics. The relative value of luciferase was also evaluated by Centro LB960 XS3 (Berthold, German).

### Biotin-labeled probe pull-down assay

A biotinylated circHERC4 probe was designed and synthesized by IGE Biotech Co. Ltd. (Shanghai, China), with oligonucleotide probes as negative controls. Approximately 1 × 10^7^ cells were harvested, lysed and sonicated. Then the lysate was incubated with biotinylated probes for 2 h and antibiotic streptomycin magnetic beads (Invitrogen, CA, USA) for 4 h. After the incubation, the magnetic beads were washed and the pull-down complexes were extracted with Trizol reagent and analyzed by qRT-PCR. Probe sequence of RNA pull-down is shown in Additional file 1: Table S4.

### Fluorescence in situ hybridization (FISH)

The specific fluorescently labeled circHERC4 and miR-556-5p FISH probes were designed and synthesized by Servicebio (Guangzhou, China). The probe sequences are listed in Additional file 1: Table S5. The FISH experiment was performed according to the manufacturer’s instructions. All images were acquired on Nikon A1Si Laser Scanning confocal Microscope (Nikon Instruments Inc., Japan). Data analyses were performed by estimating the number of fluorescent cells.

### In vivo studies

Four-week-old female BALB/c nude mice were purchased from the Experimental Animal Center, Sun Yat-Sen University (Guangzhou, China). The animal studies were approved by the Institutional Animal Care and Use Committee of SYSUCC. HCT116 cells (1 × 10^6^) stably expressing sh-NC, sh-circHERC4-1, sh-circHERC4-2 or SW480 cells (1 × 10^7^) stably expressing pLCDH-vector or pLCDH-circHERC4 were established. Mice were randomly divided into seven groups (*n* = 6): sh-NC, sh-circHERC4-1, sh-circHERC4-2; vector, circHERC4 OE, circHERC4 OE + CTBP2 siRNA, circHERC4 OE + miR-556-5p mimic. In the subcutaneous tumor formation experimental group, the stable cells that were injected into the upper back of nude mice were collected and resuspended in PBS (100 μL) and matrigel substrate (100 μL). The animals were killed 35 days after injection, and the tumors were collected to measure the tumor volume every 7 days. The tumor volume was calculated using the following formula: volume (mm^3^) = length × width^2^/2. The tumors were fixed in 4% paraformaldehyde for 24 h and then paraffin-embedded. For the tail vein injection experimental group, the stable cells that were injected into the tail vein were collected and resuspended in PBS (100 μL). What is more, for the rescue experiment, in vivo CTBP2 siRNA and in vivo miR-556-5p mimics were designed by RIBOBIO (Guangzhou, China). In the subcutaneous tumor formation experimental group, siRNA and mimics were injected into the subcutaneous tumor site 1 nmol per mouse and twice per weekly. For the tail vein injection experimental group, siRNA and mimics were injected into the tail vein of nude mice 5 nmol per mouse and twice per weekly. The animals were killed 60 days after injection. The liver and lung were harvested and fixed in 4% paraformaldehyde for 24 h, and then paraffin-embedded. Then HE staining was performed to analyze the formation of metastasis.

### HE and Immunohistochemistry (IHC)

The tissues were fixed in 10% formalin, embedded and cut into 4-μm-thick slices. The slices were dewaxed in xylene, rehydrated in a graded series of alcohols, then incubated with 3% hydrogen peroxide solution for 10 min to block endogenous peroxidase activity, washed with PBS for 5 min and blocked with 3% BSA solution at room temperature for 30 min. Antigen was retrieved by heating the slices at 100 °C for 30 min in EDTA solution. Next, the slices were incubated with rabbit monoclonal antibody against human CTBP2 (1: 500, #13256, CST), E-cadherin (1:200, #14472, CST) and Ki-67 (1:200, #9449, CST) at 4 °C overnight. After PBS washing, the slices were incubated with goat anti-rabbit secondary antibodies. Real EnVision Detection System (Dako, Denmark) was used as the chromogen, and hematoxylin was applied as the nuclear counterstain. Finally, after dehydration and clearing, the slices were mounted by neutral balsam. Images were captured under a microscope with a core data acquisition system (NIS elements 4.0; Nikon Corporation, Tokyo, Japan). The evaluation procedure was based on staining intensity and distribution of staining cells as follows: staining intensity for CTBP2, E-ca and Ki67 was scored as 0 (negative), 1 (weak), 2(moderate) and 3 (strong). Staining extent was scored as 0 (0), 1 (≤ 10%), 2 (10–50%), 3(50–80%), 4 (> 80%). The product of staining intensity and extent scores was used as IHC scores. The sections were reviewed by two pathologists.

### Statistical analysis

Statistical data were analyzed using SPSS 22.0 (IBM Corp., Armonk, NY, USA), R 4.0.5 and GraphPad Prism 8.0. Each experiment was repeated at least three times, and the data were shown as mean ± standard deviation in the bar charts. The differences among multiple groups were performed with the Student t-tests or one-way ANOVAs. The association of the expression of circHERC4 with the patient’s clinicopathological parameters was performed with the Chi-squared test. Overall survival (OS) curves were analyzed with the Kaplan–Meier method and compared by the log rank test. *P* values of < 0.05 were considered significant.

## Results

### Identification and characteristics of circHERC4 in CRC

**Fig. 1:**
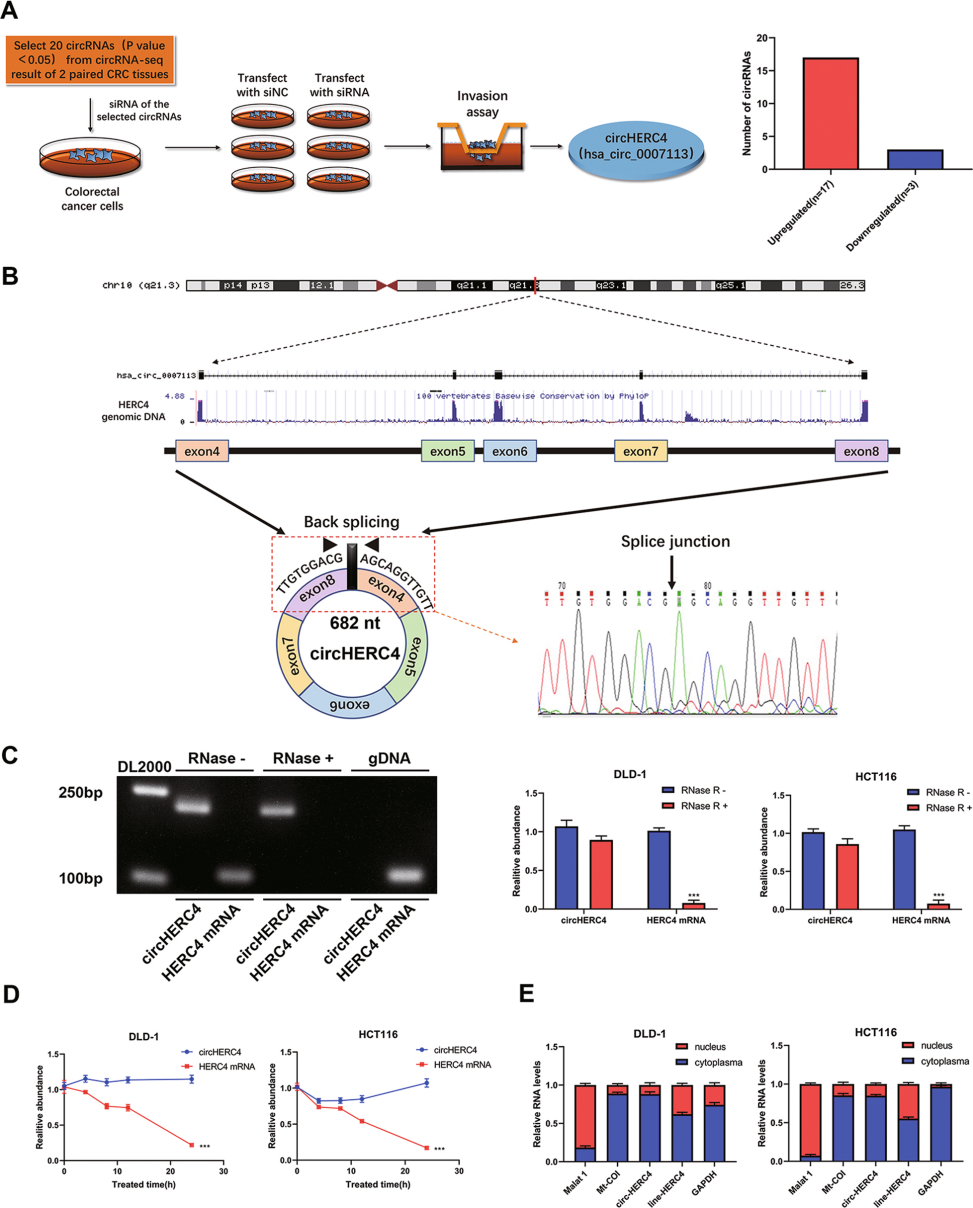
Identification and characterization of circHERC4 in CRC cells. **a** Pattern diagram of the method to screen circHERC4. **b** Explanation of the illustrated genomic loci of HERC4, and the verification strategy for the circular exon 4–8 (circHERC4). Sanger sequencing following PCR was used to show the “head-to-tail” splicing of circHERC4. **c** Relative RNA level of circHERC4 and linear HERC4 treated with RNase R, ***, *P* < 0.001. **d** Relative RNA level of circHERC4 and linear HERC4 in different time point treated with actinomycin D, ***, *P* < 0.001. **e** Nuclear and cytoplasmic fractions were isolated. CircHERC4 was mainly localized in cytoplasm. Malat 1 was substantially expressed in nucleus and Mt-COI was mostly contained in cytoplasm, which served as cytoplasmic and nuclear RNA marker

Firstly, two paired CRC tissues and matched adjacent non-tumor tissues were selected to carry out the RNA-seq to analyze any alternative expression profiles of circRNAs. Among them, 20 circRNAs that have significant difference expression (fold change ≥ 10.0 and *P* < 0.05) in CRC tissues were chosen (17 upregulated circRNAs, 3 downregulated circRNAs). To select metastasis-relative circRNAs, siRNAs of these selected circRNAs were transfected into CRC cells and a transwell invasion assay was applied to screen out the functional circRNAs. According to the above results, circHERC4 (has_circ_0007113) was found to be the most significant metastatic driving circRNA which was overexpressed in CRC (Fig. 1a). CircHERC4 (chr10: 69,785,302–69,804,320) arises from the HERC4 gene and consists of the head-to-tail splicing of exon 4–8 (682 bp) as reported in circBase. We designed a divergent primer to amplify the back-spliced form of HERC4, and Sanger sequencing was used to certify the existence of spliced junctions in circHERC4 (Fig. 1b). In DLD-1 cells, circHERC4 could only be amplified in cDNA but not gDNA, eliminating artifacts caused by genomic rearrangement (Fig. 1c). Furthermore, we performed RNase R treatment and actinomycin D assay to confirm the stability of circHERC4. The level of HERC4 mRNA was downregulated significantly after the treatment of RNase R and actinomycin D, while the level of circHERC4 remained untouched (Fig. 1c, d). Moreover, through qRT-PCR after nucleocytoplasmic separation, we found that circHERC4 was chiefly located in cell cytoplasm (Fig. 1e).

### Overexpression of circHERC4 but not HERC4 mRNA is correlated with poor patient prognosis in CRC

**Fig. 2:**
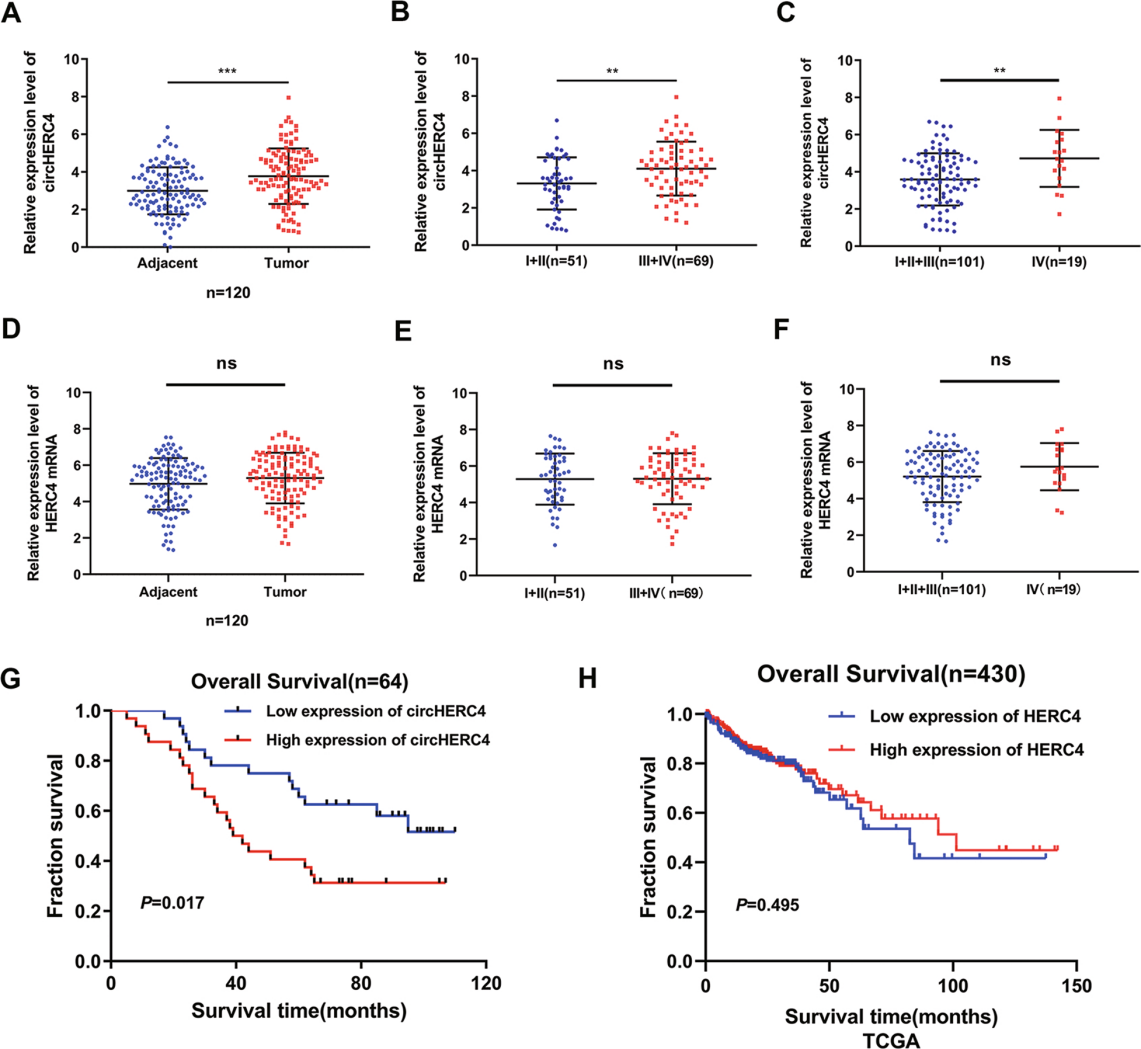
CircHERC4 was overexpressed in CRC tissues and associated with metastasis. **a** Relative RNA level of circHERC4 in CRC tissues and paired adjacent normal tissues, ***, *P* < 0.001. **b** Relative RNA level of circHERC4 in CRC tissues with lymphatic metastasis compared to those without lymphatic metastasis, **, *P* < 0.01. **c** Relative RNA level of circHERC4 in CRC tissues with distant metastasis compared to those without distant metastasis, **, *P* < 0.01. **d** Relative RNA level of HERC4 mRNA in CRC tissues and paired adjacent normal tissues, ns: no significance. **e** No significant changes were found in HERC4 mRNA expression between CRC tissues with and without lymphatic metastasis. ns: no significance. **f** No significant changes were found in HERC4 mRNA expression between CRC tissues with and without distant metastasis. ns: no significance. **g** Kaplan–Meier analysis of the association between circHERC4 expression and CRC patient overall survival (OS) in the 64-patient cohort. **h** Data from TCGA showed no significant association between HERC4 mRNA expression and CRC patient OS, ns: no significance

To further characterize the oncogenic phenotype of circHERC4 in CRC, qRT-PCR was performed to examine the expression of circHERC4 in 120 pairs of tissue samples (120 CRC tissues and 120 matched adjacent normal tissues). Notably, the result suggested that the expression of circHERC4 was significantly increased in CRC patients (*P* < 0.001) (Fig. 2a). In addition, the high expression of circHERC4 was positively associated with lymphatic metastasis (*P* < 0.01) (Fig. 2b) and distant metastasis, principally liver metastasis (*P* < 0.01) (Fig. 2c). The correlation analysis in 120 cases of CRC patients with clinical characteristics revealed that circHERC4 expression was associated with the pathological stage and histological grade using Chi-squared test (Table 1). We were also concerned whether the overexpression of circHERC4 was caused by the upregulation of host HERC4 mRNA in CRC. However, no significant increase of HERC4 mRNA was found in our 120 cohort CRC tissues (Fig. 2d) and the same tendency was found in CRC tissues derived from TCGA (Additional file 2: Fig. S1A, B). Likewise, HERC4 mRNA was not correlated with lymphatic metastasis (Fig. 2e and Additional file 2: Fig. S1C) and liver metastasis (Fig. 2f and Additional file 2: Fig. S1D). So we supposed that the overexpression of circHERC4 was regulated post-transcriptionally in CRC. Furthermore, Kaplan–Meier survival analysis revealed that patients with high circHERC4 expression were associated with poorer overall survival (*P* < 0.05) (Fig. 2g). HERC4 mRNA expression level had no correlation to the prognosis of the patient (Fig. 2h). Taken together, these results suggest that circHERC4, but not its host HERC4 mRNA, is significantly upregulated in CRC tissues and higher circHERC4 expression may imply a poor prognosis.

**Table 1:** Clinical characteristics of CRC patients with low circHERC4 expression and high circHERC4 expression

	Cases	circHERC4 expression		*p* value
		Low	High	
Age				0.456
< 60	48	26	22	
≥ 60	72	34	38	
Gender				0.267
Male	70	38	32	
Female	50	22	28	
T stage				0.023
T1–T3	76	44	32	
T4	44	16	28	
Lymph node metastasis				0.006
Absent	51	33	18	
Present	69	27	42	
Distant metastasis				0.006
Absent	101	56	45	
Present	19	4	15	
Total	120	60	60	

### CircHERC4 behaves as an oncogene and promotes proliferation, migration and invasion of CRC in vitro and in vivo

**Fig. 3:**
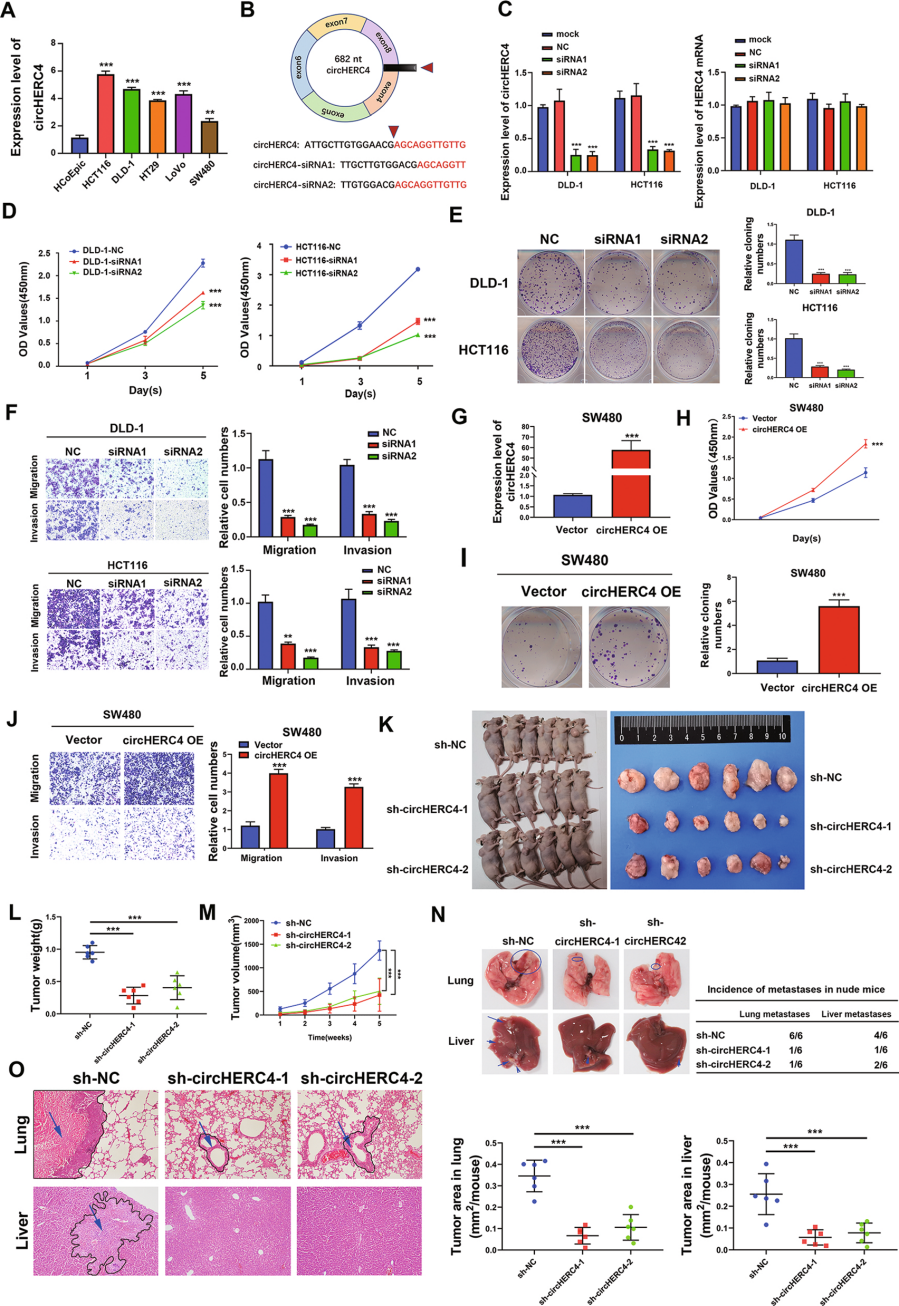
CircHERC4 acted as an oncogene and regulated proliferation, migration and invasion in *vitro* and in *vivo*. **a** Basic expression of circHERC4 in HCoEpiC, HCT116, DLD-1, HT29, LoVo and SW480 cell lines, **, *P* < 0.01, ***, *P* < 0.001. **b** The siRNA was designed to target the back-splice junction site of circHERC4. **c** After the transfection of siRNA of circHERC4, qPCR was used to detect expression of circHERC4, ***, *P* < 0.001. The expression of HERC4 mRNA was not affected by siRNA of circHERC4. **d** CCK8 assays showed that knockdown of circHERC4 induced the repression of viability of DLD-1 and HCT116 cells. **e** Colony formation assays revealed that silencing of circHERC4 could inhibit the proliferation ability in DLD-1 and HCT116 cells, ***, *P* < 0.001. **f** Transwell assays demonstrated that migration and invasion abilities of DLD-1 and HCT116 were impaired by siRNA of circHERC4, **, *P* < 0.01, ***, *P* < 0.001. **g** qPCR was performed to detect circHERC4 after overexpression of circHERC4 in SW480 cells, ***, *P* < 0.001. **h** CCK8 assays showed that overexpression of circHERC4 upregulated viability of SW480 cells. **i** Colony formation assays revealed that overexpression of circHERC4 could promote the proliferation ability in SW480 cells, ***, *P* < 0.001. **j** Migration and invasion abilities of SW480 were enhanced by overexpression of circHERC4, ***, *P* < 0.001. **k** Images of xenografts tumor (six mice per group) in nude mice. **l, m** Comparison of the tumor weight and volume in sh-circHERC4-1, sh-circHERC4-2 and sh-NC HCT116 group, ***, *P* < 0.001. **n** Images and statistics of visible nodules on the lung and liver surface. **o** Representative images and HE staining of lung and liver harvested from the tail vein injection group showed that incidence and area of metastasis nodules in experimental groups was significantly lower than negative control group, ***, *P* < 0.001

To determine the functional role of circHERC4, we detected the expression of circHERC4 in human normal colonic epithelial cells, HCoEpic and CRC cells (HCT116, DLD-1, HT29, LoVo and SW480). We found the expression of circHERC4 was significantly upregulated in CRC cells, among which the circHERC4 expression was relatively high in DLD-1 and HCT116 cells and relatively low in SW480 cells compared with HCoEpic cells (Fig. 3a). Therefore, HCT116, DLD-1 and SW480 cells were selected for further study. We designed siRNAs targeting the back-spliced sequence of circHERC4 (Fig. 3b). DLD-1 and HCT116 cells were transfected with circHERC4 siRNAs which successfully silenced the circHERC4 but not HERC4 mRNA as confirmed by qRT-PCR analysis (Fig. 3c). According to the CCK-8 assay, silencing of circHERC4 significantly inhibited the viability of DLD-1 and HCT116 cells (Fig. 3d). What is more, colony formation assay revealed that proliferative abilities of DLD-1 and HCT116 cells were also repressed in circHERC4 downregulated group compared to negative control (NC) group (Fig. 3e). Furthermore, transwell assay revealed that silencing of circHERC4 restrained the migration and invasion abilities of DLD-1 and HCT116 cells (Fig. 3f). In contrast, a circHERC4-overexpressing plasmid was successfully constructed and transfected into SW480 cells, and significant increase of circHERC4 was observed (Fig. 3g). It was indicated that upregulation of circHERC4 significantly promoted the proliferation, migration and invasion abilities of CRC cells in vitro (Fig. 3h–j).

Moreover, our xenograft and metastasis mouse models also supported the view that circHERC4 played an oncogenic role in vivo. In the subcutaneous neoplasia model, circHERC4 silencing (sh-circHERC4-1 and sh-circHERC4-2) and negative control (sh-NC) HCT116 cells were subcutaneously injected into BALB/c nude mice. The tumor volumes were measured every 7 days, and the tumors were allowed to grow for 35 days. The results revealed that the growth rate and tumor weight of tumors in the sh-circHERC4 groups were significantly inhibited compared to the negative control group (Fig. 3k–m). As to the metastasis mouse model, the three groups of HCT116 cells described previously were injected into the tail vein of nude mice. After growing for 60 days, the mice were killed, and the lungs and livers were harvested for HE staining to detect the formation of metastasis. The metastatic nodules were found in the lung and liver (Fig. 3n). The incidence of lung and liver metastases was significantly decreased in sh-circHERC4 groups, in contrast to that in negative control group (Fig. 3n). HE staining further showed that the area of metastatic nodules was extremely high in sh-NC group, while the area of metastatic nodules was significantly lower in sh-circHERC4 groups (Fig. 3o, Additional file 3: Fig. S2 and Additional file 4: Fig. S3). All these above data confirmed that circHERC4 could promote the progression, especially metastasis of CRC in vitro and in vivo.

### CircHERC4 directly binds to miR-556-5p in CRC cells

**Fig. 4:**
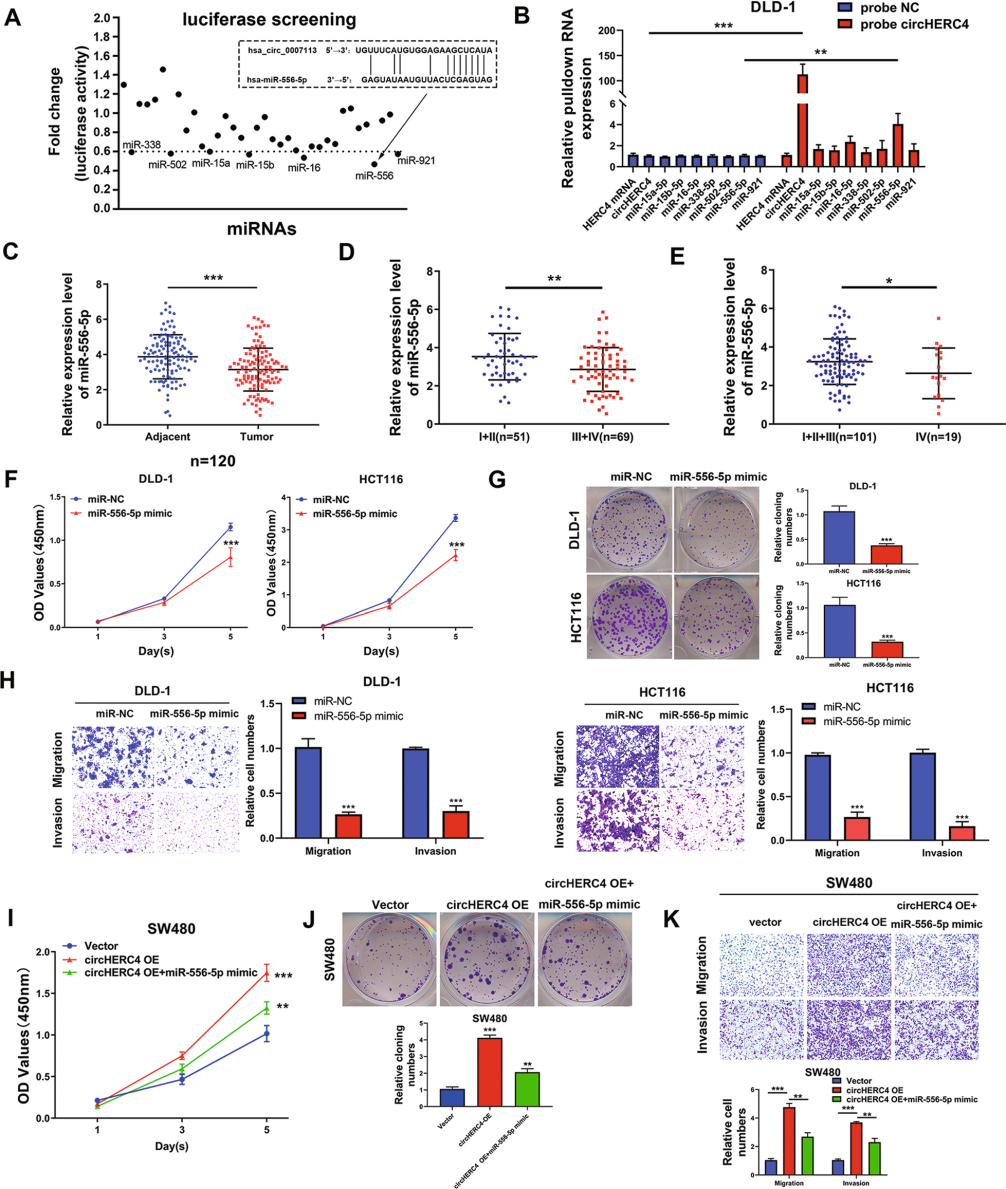
CircHERC4 accelerated CRC cell proliferation, migration and invasion by interacting with miR-556-5p. **a** Luciferase report experiment was used to pre-screen microRNAs that could bind to circHERC4. **b** RNA pull-down assays demonstrated that miR-556-5p was significantly enriched in the circHERC4 probe compared to NC probe, **, *P* < 0.01, ***, *P* < 0.001. **c** Expression of miR-556-5p was significantly downregulated in CRC tissues compared to matched adjacent normal tissues, ***, *P* < 0.001. **d** Expression of miR-556-5p in CRC tissues with lymphatic metastasis was significantly lower than those CRC tissues without lymph node metastasis, **, *P* < 0.01. **e** Relative RNA level of miR-556-5p was higher in the CRC tissues without distant metastasis compared to the CRC tissues with distant metastasis, *, *P* < 0.05. **f** CCK8 assays demonstrated that overexpression of miR-556-5p could inhibited the viabilities of DLD-1 and HCT116 cells, ***, *P* < 0.001. **g** Colony formation assays showed that overexpression of miR-556-5p repressed the formation of colonies in DLD-1 and HCT116 cells, ***, *P* < 0.001. **h** Transwell assays revealed that overexpression of miR-556-5p restrained migration and invasion abilities of DLD-1 and HCT116 cells, ***, *P* < 0.001. **i–k** Rescue experiment showed that miR-556-5p could partially reverse the oncogenic function of circHERC4 in proliferation, migration and invasion, **, *P* < 0.01, ***, *P* < 0.001

CircHERC4 being predominantly located in the cytoplasm of CRC cells suggests that it may function post-transcriptionally. So we predicted candidate-binding miRNAs through StarBase (http://starbase.sysu.edu.cn/index.php) and Circinteractome (https://circinteractome.nia.nih.gov/). Among these diverse miRNAs, thirty-six miRNAs were preliminarily screened out. To identify whether these miRNAs could bind to circHERC4, we performed a luciferase screening with a miRNA mimic library. We constructed a circHERC4 fragment and inserted it into the downstream of the luciferase reporter gene in the psiCHECK-2 plasmid. Then miRNA mimics were co-transfected with the luc-circHERC4 reporter plasmid into 293T cells. Compared with the scrambled control, seven miRNAs could reduce the luciferase activity by at least 40%, and miR-556-5p was shown to have the greatest luciferase activity inhibiting effect in dual-luciferase reporter assay (Fig. 4a). To confirm this prediction, biotinylated circHERC4 probes targeting the junction site and scrambled oligo probes were designed and applied to perform RNA pull-down assay in DLD-1 cells. The qPCR assay following the RNA pull-down assay showed that circHERC4, but not the host HERC4 mRNA, could be enriched significantly. All the seven miRNAs which had the luciferase activity inhibiting effect were tested in circHERC4 pull-down fraction by qPCR assay. The enrichments of miR-556-5p in the circHERC4 pull-down fraction were significantly higher than NC group and other miRNAs (Fig. 4b). These results suggested that miR-556-5p was directly bound to circHERC4, and might participate in the function of circHERC4 in CRC.

### Inhibition of miR-556-5p mediates the oncogenic function of circHERC4 and activates the Notch signaling pathway in CRC

On account of the fact that there were no studies about miR-556-5p in CRC, we first confirmed the expression pattern in our clinical CRC samples (*n* = 120) by qPCR assay. We validated that miR-556-5p was frequently downregulated in CRC tissues compared with the matched adjacent normal tissues (Fig. 4c). We further analyzed the expression levels of miR-556-5p in CRC patients with or without metastasis, and the result revealed that the expression levels of miR-556-5p were much lower in the patients with lymphatic metastasis (Fig. 4d) or distant metastasis (Fig. 4e). The expression of miR-556-5p was also evaluated in the CRC patients as mentioned before using a Chi-squared test, and the results showed that patients with positive lymphatic metastasis had low levels of miR-556-5p, while other features had no significant correlation with the expression of miR-556-5p (Table 2). Then we analyzed the correlation between circHERC4 and miR-556-5p in 120 CRC samples. The result implied that miR-556-5p was negatively related to circHERC4 (Additional file 5: Fig. S4). Furthermore, these consequences implied that miR-556-5p was markedly inhibited in CRC and might act as a tumor suppressor. To explore the functional role of miR-556-5p, we transfected miR-556-5p mimics into DLD-1 and HCT116 cells. All the results displayed that miR-556-5p could significantly inhibit the ability of proliferation, migration and invasion of CRC cells (Fig. 4f–h). Moreover, rescue experiments were conducted by co-transfecting miR-556-5p mimics and circHERC4 overexpression vectors in SW480. Remarkably, miR-556-5p mimics could partially restore circHERC4 overexpression-enhanced SW480 cells’ proliferation, migration and invasion ability (Fig. 4i–k). Taken together, all these results provided the evidence that miR-556-5p exhibited the anticancer role and partially mediated the oncogenic function of circHERC4 by direct binding in CRC.

**Table 2:** Clinical characteristics of CRC patients with low miR-556-5p expression and high miR-556-p expression

	Cases	miR-556-5p expression		*p* value
		Low	High	
Age				0.264
< 60	48	27	21	
≥ 60	72	33	39	
Gender				0.139
Male	70	31	39	
Female	50	29	21	
T stage				0.705
T1–T3	76	37	39	
T4	44	23	21	
Lymph node metastasis				0.006
Absent	51	33	18	
Present	69	27	42	
Distant metastasis				0.211
Absent	101	48	53	
Present	19	12	7	
Total	120	60	60	

**Fig. 5:**
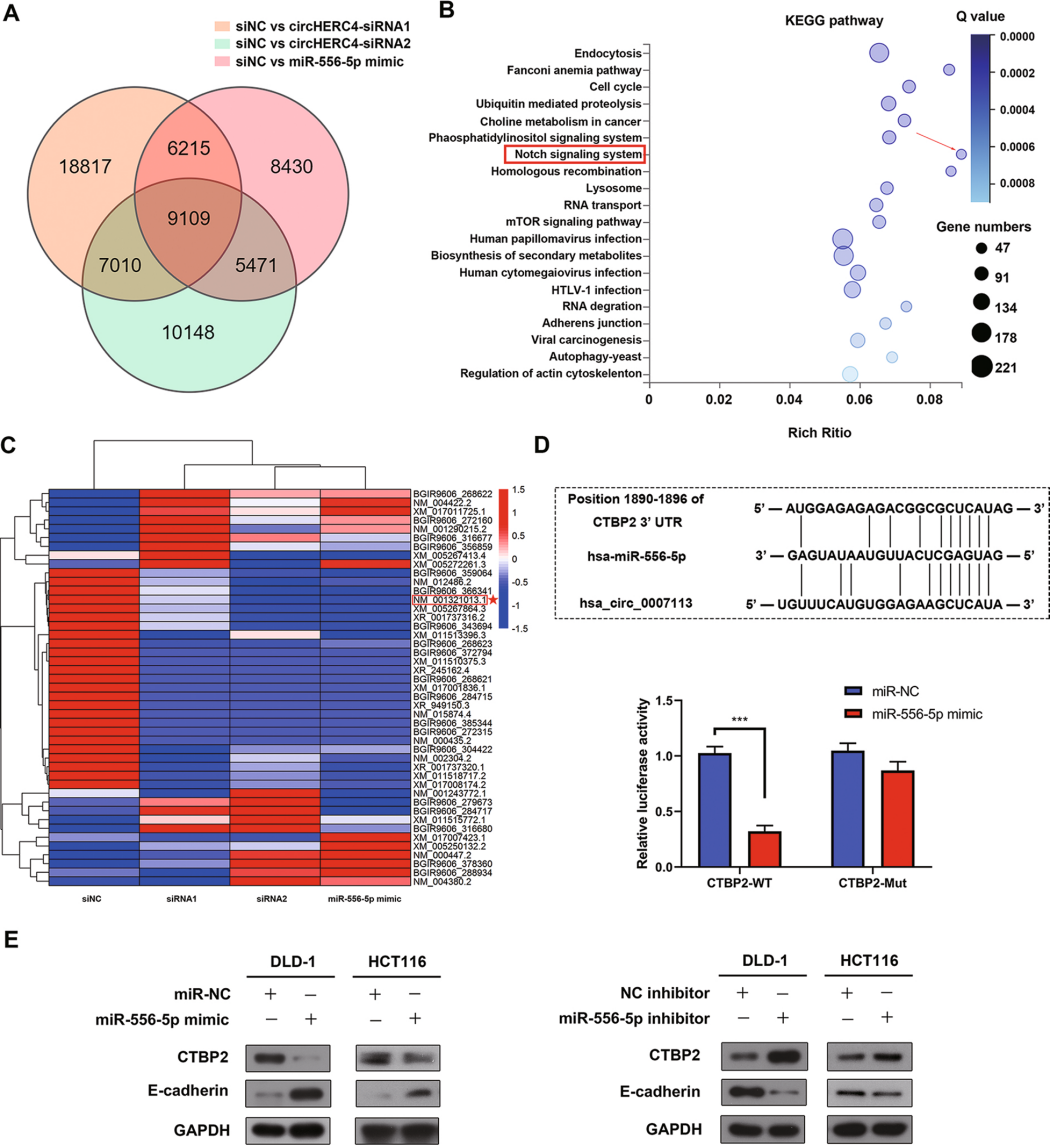
CTBP2 was a target gene of miR-556-5p. **a** RNA-seq was used to predict the target gene of miR-556-5p in CRC; the venn diagram showed that 9109 transcripts were changed together in three groups. **b** KEGG pathway analysis demonstrated that Notch signaling system was involved and might be the downstream of circHERC4 and miR-556-5p. **c** Heat map was performed to analyze the changed genes that were contained in the Notch signaling system. NM 001321013.1: CTBP2. **d** Upper, predicted binding sites between miR-556-5p and CTBP2. Lower, luciferase reporter experiment in DLD-1 co-transfected with miR-556-5p mimics and psi-check2-mutant type CTBP2 plasmid compared to psi-check2 wild-type CTBP2 plasmid, ***, *P* < 0.001. **e** Left, Western blotting showed that CTBP2 was downregulated and E-cadherin was upregulated when miR-556-5p was overexpressed. Right, knockdown of miR-556-5p inhibited CTBP2 and activated E-cadherin

However, the downstream signaling pathway might be affected by the circHERC4/miR-556-5p axis was unknown. So, we performed the RNA-seq after silencing circHERC4 or overexpressing miR-556-5p. According to the Venn diagram (Fig. 5a), we screened out 9109 transcripts that were altered significantly both in circHERC4 silencing and miR-556-5p overexpressing DLD-1 cells. KEGG pathway enrichment analyses showed that these 9109 transcripts were enriched in many tumor-associated signaling pathways, especially the Notch signaling pathway which was widely known as activator of colon carcinogenesis (Fig. 5b). We hypothesized that circHERC4 might promote cell metastasis by activating the Notch signaling pathway which might be blocked by miR-556-5p.

### CTBP2 is a target of miR-556-5p and exerts an oncogenic role by inhibiting the expression of E-cadherin in CRC

We further explored the effector of the Notch signaling pathway targeted by miR-556-5p. A heat map was used to show that most genes contained in the Notch signaling pathway were downregulated by silencing of circHERC4 and overexpressing miR-556-5p (Fig. 5c). Combining with the bioinformatic analysis, CTBP2 was predicted to be a target of miR-556-5p (Fig. 5d). To further confirm this hypothesis, a luciferase assay was performed to verify the interaction between miR-556-5p and CTBP2. CTBP2 3’UTR fragment containing the wild-type and mutant binding sites with miR-556-5p was cloned into the luciferase reporter plasmid psiCHECK2 and co-transfected with miR-556-5p mimic or miR-NC mimic into DLD-1 cells. The results showed that overexpression of miR-556-5p significantly diminished the luciferase activity of the vector including the wild-type binding site but not the mutant binding site (Fig. 5d). Moreover, we performed the Western blot assay to prove that miR-556-5p could markedly inhibit the protein level of CTBP2, but rescued the expression of E-cadherin which was reported to be the target of CTBP2 [26], and the result is shown in Fig. 5e.

**Fig. 6:**
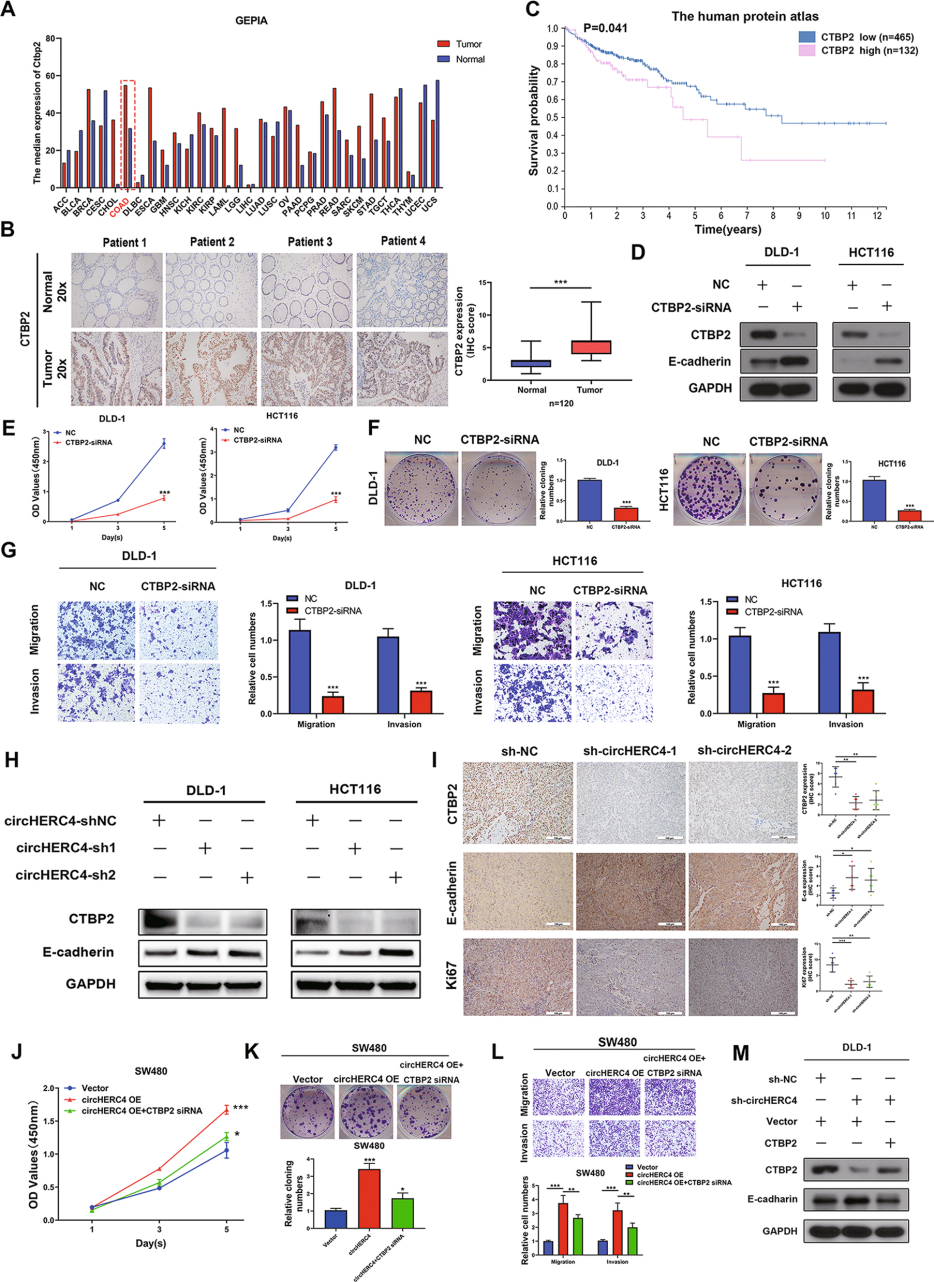
CTBP2 was overexpressed in CRC tissues and could promote the progression of CRC cells. **a** GEPIA database showed that CTBP2 was significantly upregulated in CRC tissues. **b** Kaplan–Meier analysis of survival probability from The Human Protein Atlas database showed that higher CTBP2 expression was associated with shorter survival time. **c** IHC staining of CTBP2 in clinical CRC tissues and matched adjacent normal tissues, *n* = 120, ***, *P* < 0.001. **d** Efficiency of CTBP2 siRNA was detected by Western blotting. **e–g** CCK8, colony formation and transwell assays presented that knockdown of CTBP2 could significantly repress the proliferation, migration and invasion of DLD-1 and HCT116 cells, ***, *P* < 0.001. **h** Western blotting showed that CTBP2 was downregulated and E-cadherin was upregulated when circHERC4 was silenced. **i** IHC staining was used to detect CTBP2, E-cadherin and Ki67 expression in sh-NC, sh-circHERC4-1 and sh-circHERC4-2 HCT116 group. **j–l** Schemes revealed that oncogenic function of circHERC4 was rescued by downregulation of CTBP2, *, *P* < 0.05, **, *P* < 0.01, ***, *P* < 0.001. **m** Western blotting was performed to reveal that overexpression of CTBP2 could partially rescue the effect of silenced circHERC4 on the expression of E-cadherin

CTBP2 was hinted to constitute an essential facet of tumor development in colorectal cancer [29]. We searched the GEPIA database (http://gepia.cancer-pku.cn/index.html) and found that the median expression of CTBP2 protein was upregulated in colorectal cancer tissues compared with adjacent normal tissues (Fig. 6a). Next, we investigated the expression of CTBP2 mRNA in our 120 cohort CRC samples. As shown in Additional file 6: Fig. S5A, CTBP2 mRNA level was overexpressed in tumor tissues compared to adjacent non-cancerous tissues. What is more, CTBP2 and metastasis showed potential of correlation (Additional file 6: Fig. S5B, C). The same result that high expression of CTBP2 was common in the CRCs was also explored in our IHC assay (*n* = 120) (Fig. 6b). Furthermore, a Kaplan–Meier survival analysis from The Human Protein Atlas (https://www.proteinatlas.org/) evaluated that high expression of CTBP2 was significantly associated with poorer survival probability in CRC (*P* < 0.05) (Fig. 6c). We constructed and transfected CTBP2 siRNA into DLD-1 and HCT116 cells, and the declined expression level of E-cadherin protein was confirmed by Western blot assay (Fig. 6d). Compared with the negative control (NC) group, silencing of CTBP2 significantly inhibited the expression of the proliferation, migration and invasion ability of DLD-1 and HCT116 cells (Fig. 6e–g). Together these results provided important insights into the fact that CTBP2 was a target gene of miR-556-5p and its overexpression mediated the tumor metastasis through inhibiting E-cadherin in CRC.

### Silencing of CTBP2 rescues the effect induced by overexpression of circHERC4

**Fig. 7:**
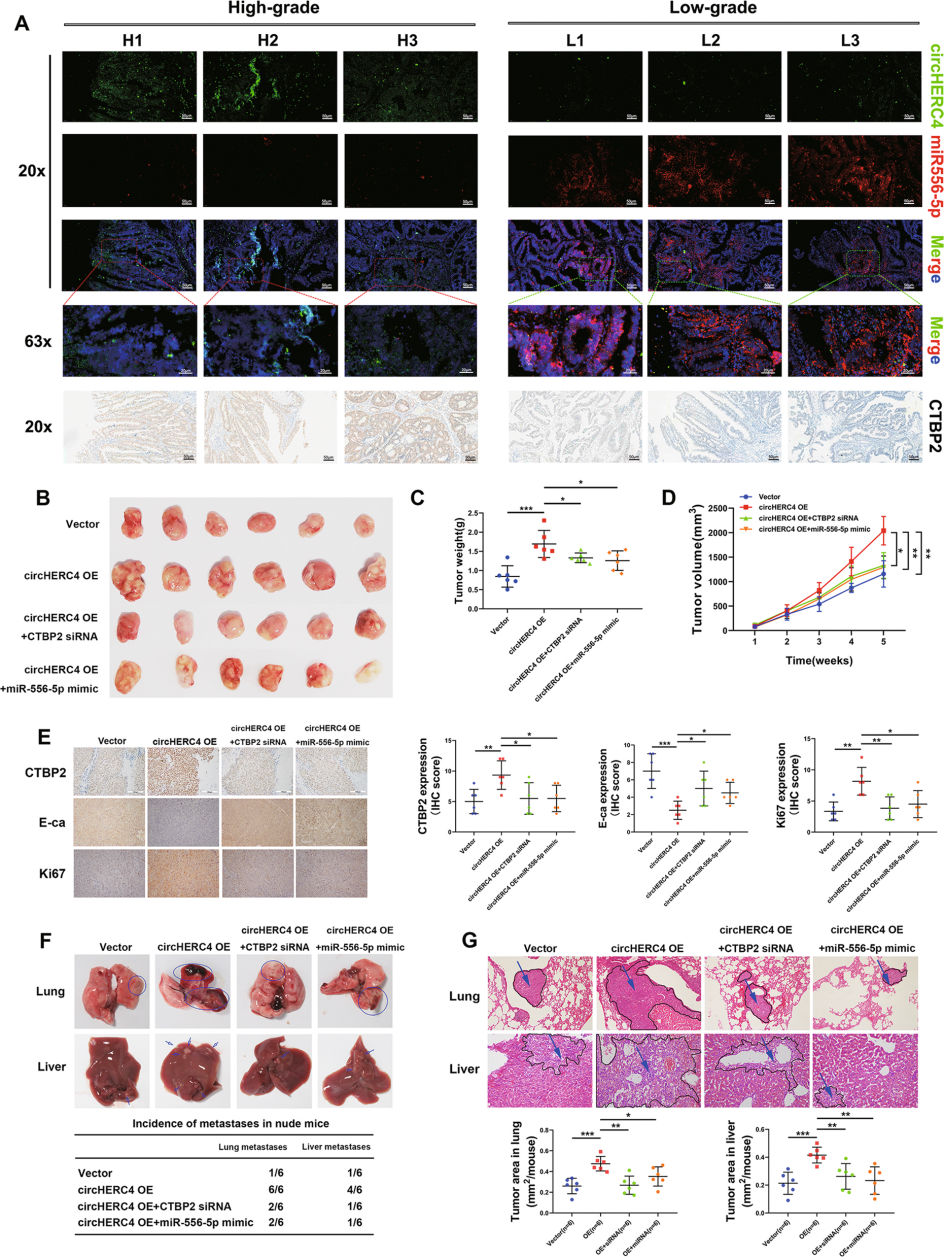
CTBP2 was regulated by circHERC4/miR-556-5p interaction. **a** Representative image of the co-expression of circHERC4, miR-556-5p and CTBP2 in CRC tissues divided into high-grade group and low-grade group according to the clinical stage. **b** Representative images of subcutaneous xenograft tumors formed by SW480 cells stably transfected as indicated in nude mice. **c**, **d** Tumor weights (**c**) and tumor volumes (**d**) were measured for each group (*n* = 6 per group). *, *P* < 0.05, **, *P* < 0.01, ***, *P* < 0.001. **e** IHC staining of CTBP2, E-ca and Ki67 in each group. Overexpression of circHERC4 could upregulate CTBP2 and Ki67 and downregulate E-ca expression; the effect could be rescued by inhibiting of CTBP2 or overexpression of miR-556-5p. *, *P* < 0.05, **, *P* < 0.01, ***, *P* < 0.001. **f** Images and statistics of visible nodules on the lung and liver surface. **g** Representative images of HE staining analysis of metastatic nodules in lungs and livers. *, *P* < 0.05, **, *P* < 0.01, ***, *P* < 0.001

We had confirmed that circHERC4 could block miR-556-5p, so we further verified whether circHERC4 manipulated the expression of miR-556-5p target, CTBP2. Lv-circHERC4-shRNAs were transfected into DLD-1 and HCT116 cells separately. We found that CTBP2 protein level decreased significantly when circHERC4 was knocked down. Inversely, E-cadherin, the downstream target of CTBP2, was increased significantly under these conditions (Fig. 6h). IHC staining showed that the expression of CTBP2 (Additional file 7: Fig. S6) was significantly decreased in the tumor tissues of the sh-circHERC4 groups compared to the sh-NC group in xenograft models. At the same time, the expression level of E-cadherin (Additional file 8: Fig. S7) was induced and Ki-67 (Additional file 9: Fig. S8) was downregulated sharply after circHERC4 was silenced (Fig. 6i). To further investigate whether circHERC4 exerts its oncogenic effect on CRC by inducing CTBP2 expression, we performed a rescue experiment to examine the functional interaction between circHERC4 and CTBP2. We found that the proliferative, migratory and invasive abilities of overexpressed circHERC4 cells transfected with CTBP2 siRNA mimics obviously decreased compared with those of SW480 cells transfected with empty vector or siNC mimics (Fig. 6j–l), suggesting that the oncogenic function of circHERC4 could be partially reversed by eliminating the expression of CTBP2. Western blotting was performed to reveal that CTBP2 could partially rescue the effect of circHERC4 on the expression of E-cadherin (Fig. 6m). Moreover, depending on the clinical stage of CRC samples, FISH was performed in ten high-grade and ten low-grade CRC samples to confirm the discovery of the circHERC4/miR-556-5p/CTBP2 axis. We found that circHERC4 and CTBP2 was overexpressed in high-grade CRC tissues while miR-556-5p was more abundant in low-grade ones (Fig. 7a and Additional file 10: Fig. S9A, B). We also analyzed the correlation between circHERC4 and miR-556-5p and found a more significant negative correlation (Additional file 10: Fig. S9C) compared with aforementioned results (Additional file 5: Fig. S4). The reasons for the difference between them might attribute to the level of clinical staging and tumor heterogeneity. Finally, an in vivo rescue experiment containing four groups (vector, circHERC4 OE, circHERC4 OE + CTBP2 siRNA, circHERC4 OE + miR-556-5p mimic) was performed. The results of xenograft subcutaneous suggested that overexpression of circHERC4 could accelerate tumor growth and this effect could be inhibited after CTBP2 siRNA or miR-556-5p mimic was injected (Fig. 7b–d). Moreover, IHC assay was performed to indicate that upregulated circHERC4 promoted CTBP2 (Additional file 11: Fig. S10) and inhibited E-ca (Additional file 12: Fig. S11), and Ki67 (Additional file 13: Fig. S12) was also found upregulated in circHERC4-overexpression group. This trend could also be partially rescued by silencing of CTBP2 or overexpression of miR-556-5p (Fig. 7e). In tail vein injection mouse model, upregulated circHERC4 promoted the formation of metastatic nodules in lung and liver, and this circHERC4-induced carcinogenic effects were obstructed when CTBP2 was downregulated or miR-556-5p was upregulated (Fig. 7f–g, Additional file 14: Fig. S13 and Additional file 15: Fig. S14). Collectively, these results demonstrated that CTBP2 could promote CRC progression and was regulated by the circHERC4/miR-556-5p axis in vitro and in vivo.

## Discussion

Recently, as sequencing technology continues to advance, increasing numbers of identified circRNAs begin to emerge on our radar [9]. Unlike mRNA, miRNA and long non-coding RNA (lncRNA), circRNAs are derived from precursor mRNA (pre-mRNA) via back-splicing. Accumulating evidences revealed that circRNA was dysregulated in several cancers, and thus, its non-canonical functions to regulate the proliferation, migration, invasion and metastasis of cancer cells made it a potential biomarker and therapeutic target [11, 21]. Dysregulated circRNAs have also been suggested to take part in drug resistance in clinical cancer therapy including standard chemotherapy and targeted therapy [22]. Moreover, we should pay more attention to recurrence-related circRNAs and make them as markers of recurrence risk of colorectal cancer [30]. Nevertheless, the molecular mechanism of circRNAs in CRCs remains largely unknown. Previous studies showed that hsa_circ_0009361 could suppress colorectal cancer progression by sponging miR-582 [31]. Even more interestingly, circPPP1R12A could encode a small uncharacterized peptide circPPP1R12A-73aa and it was the circPPP1R12A-73aa, but not circPPP1R12A, that promoted the cell proliferation and metastasis of colon cancer [19]. In the present study, we performed a whole transcriptome sequencing and bioinformatics analysis to screen out the dysregulated circRNAs and demonstrated for the first time that circHERC4 was a critical circRNA that was significantly overexpressed in CRC tissues. High expression of circHERC4 was correlated with high grade, lymphatic and distant metastasis and poor prognosis.

Colorectal cancer (CRC) is one of the most common cancers in the world. About two-third of patients with CRC will develop distant metastasis at some point in time. The liver is the most common site where distant metastasis takes place, which urges us to identify metastasis-related genes and explore the molecular mechanisms resulting in CRC metastasis. In our study, we successfully identified the metastasis-related circRNA, circHERC4, is one of the highly expressed circRNAs from RNA-seq data of CRC tissues, which also promoted CRC cells metastasis in transwell models. In the further functional study, circHERC4 significantly facilitated the proliferation, migration and invasion of CRC cells. In the in vivo study, silencing of circHERC4 was confirmed to suppress tumor growth and metastasis in our xenograft and metastasis mouse models. Based on the above research, the oncogenic role, especially metastatic phenotype of circHERC4 in CRC was strongly confirmed.

The role of inhibiting miRNAs was claimed to be a major function of circRNAs mainly because of its higher stability than linear mRNAs and long non-coding RNAs [32]. In different cancer, miRNAs were found to be the crucial approach in EMT and CSCs (Cancer Stem Cells) inducing chemoresistance [14]. So the circRNAs may regulate EMT by targeting EMT- and CSC-related miRNAs. CircPSMC3 could suppress the proliferation and metastasis of gastric cancer by binding to miR-296-5p [33]. CircTADA2As suppressed the progression and metastasis of breast cancer according to target miR-203a-3p [34]. According to the genome-wide bioinformatics analysis of all circRNA candidates, we predicted that multiple miRNAs were capable of binding circHERC4 and a large proportion of them were associated with proliferation and metastasis in different cancer types. Next, through using luciferase reporter assay and biotin-labeled probe pull-down assay, the direct binding between miR-556-5p and circHERC4 was confirmed. After exploring the expression of circHERC4 and miR-556-5p in our own specimen library by qRT-PCR and FISH, miR-556-5p was shown to be downregulated in CRC tissues compared with paired adjacent normal tissues and had lower expression level in high-grade CRC samples compared with low-grade cohort; and a negative correlation was found between circHERC4 and miR-556-5p, which implied that miR-556-5p might act as a tumor suppressor. Considering that there were no studies about miR-556-5p in CRCs, we applied miR-556-5p mimics to transfect CRC cells. As expected, the overexpression of miR-556-5p suppressed the proliferation, migration and invasion of CRC cells. Subsequent “rescue” experiments confirmed that miR-556-5p could partially reverse the effect of circHERC4 suggesting that circHERC4 acts as an oncogene by inhibiting miR-556-5p in CRCs.

The interaction between circRNAs and miRNAs was always defined as “sponge” adsorption. However, this “sponge” interaction needs the “perfect” and extensive pairing between circRNAs and miRNAs in its seed region. Recently, two studies proved that the “perfect” pairing to RNAs could induce target directed miRNA degradation (TDMD) by the ubiquitin–proteasome pathway [35, 36]. In our study, the expression level of miR-556-5p was not influenced after silencing or overexpressing circHERC4 (data not shown). This is consistent with the idea that circRNAs may act as “boat” but not “sponge” to prevent its passengers from drowning and can move on to new ports [37]. The upregulated circHERC4 “loads” miR-556-5p to prevent its binding with its target oncogenes in CRC. The decline of miR-556-5p in CRC may be regulated by other signaling pathways.

To further decipher the underlying mechanism of circHERC4 and miR-556-5p in CRC, we performed RNA-seq to identify the differentially expressed genes after knockdown of circHERC4 or upregulation of miR-556-5p. Among the two significantly changed genes, combined with bioinformatics analysis, Notch signaling was involved in KEGG Pathway Analysis. Among them, CTBP2 was predicted to be targeted by miR-556-5p. CTBP2, as an oncogene which can inhibit E-ca gene expression, is frequently reported to promote the cell proliferation, migration, invasion and drug resistance in a variety of cancers such as breast cancer [38], prostate cancer [39], esophageal cancer [40] and colorectal cancer [41]. Then Western blot and IHC was applied to confirm the changed expression of CTBP2. Through our rescue experiment, knockdown of CTBP2 and overexpression of miR-556-5p was able to eliminate the circHERC4 upregulation-causing promotion on CRC progression in vitro and in vivo. Additionally, the effect of silenced circHERC4 on E-cadherin could be partially rescued by CTBP2 overexpression. So we proved that circHERC4 could suppress the active suppressor of invasion and growth of many epithelial cancers, E-cadherin, to promote the EMT in CRC, which consist with our results that circHERC4 significantly promote the cell migration and metastasis in mouse model. We acknowledge that our research provides only one regulatory mechanism of circHERC4 in CRCs. More works remain to be done for further exploration to expound other signaling methods.

## Conclusions

**Fig. 8:**
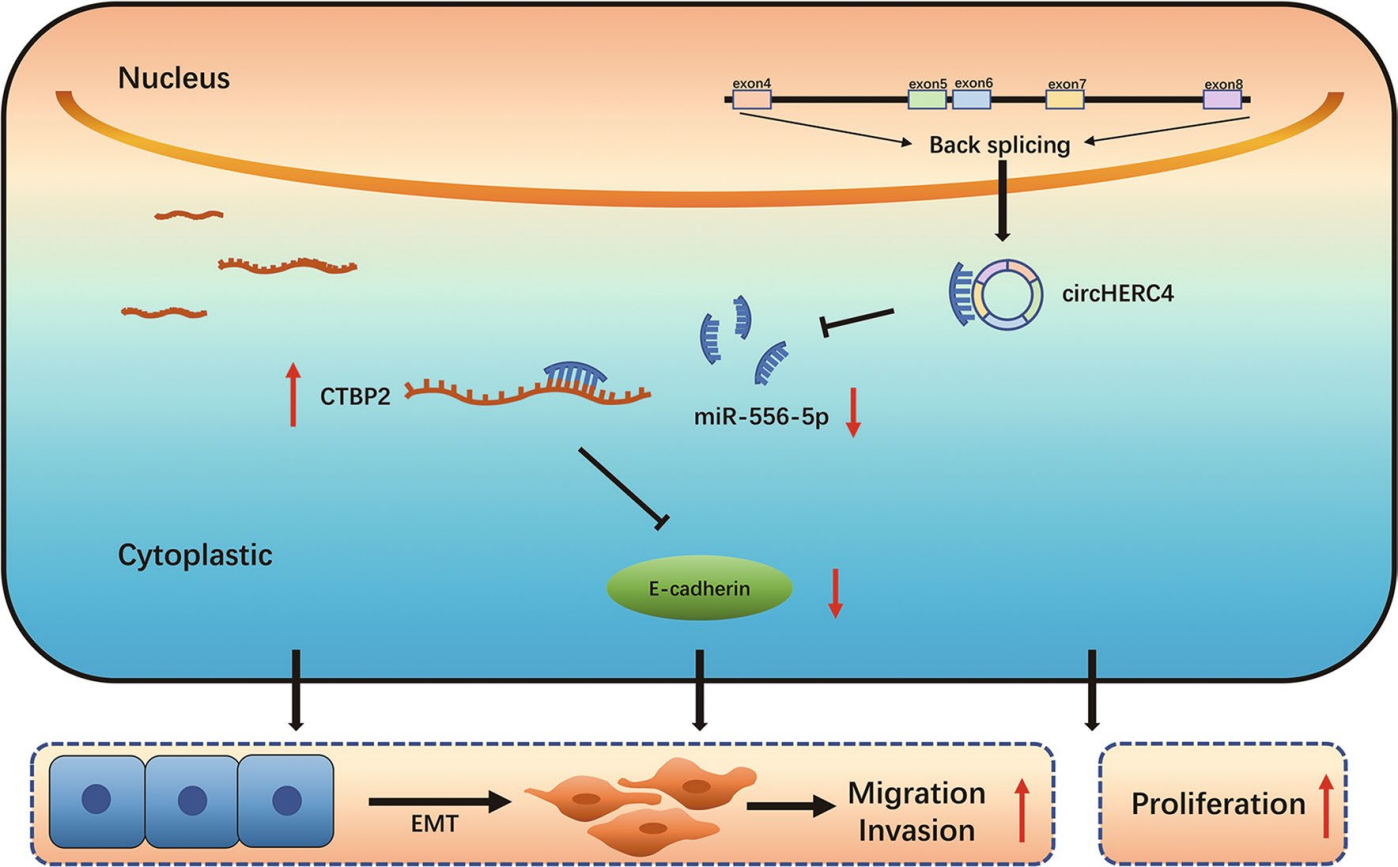
Illustration of the mechanism of circHERC4 on promoting CRC pathogenesis and metastasis via miR-556-5p/CTBP2/E-cadherin signaling pathway

In summary, our report identified that a previously unidentified oncogenic driver, circHERC4, was elevated in CRC tissues and associated with CRC proliferation, migration and invasion. Additionally, higher expression of circHERC4 in CRC patients was positively associated with metastasis and poorer survival. We proposed a mechanism that circHERC4 could block the inhibition activity of miR-556-5p on CTBP2; therefore, silencing circHERC4 could downregulate the expression of CTBP2 accompanied with the activation of E-cadherin. Importantly, our findings suggested that circHERC4 could be a potential biomarker for prognosis and silencing circHERC4 offered a novel therapeutic target for the treatment of CRC (Fig. 8).

## Supplementary Information

**Additional file 1: Table S1.** Primer sequences for the qRT-PCR. **Table S2.** microRNA primers for the qRT-PCR. **Table S3.** siRNA sequence. **Table S4.** DNA oligo sequences for pull-down assays. **Table S5**. Sequences of the FISH probes.

**Additional file 2: Figure S1.** Clinical significance of HERC4 mRNA in TCGA. **a** Relative expression level of HERC4 mRNA in CRC tissues and paired adjacent normal tissues derived from TCGA, ns: no significance. **b** HERC4 mRNA level in paired tissues in TCGA. **c** Data from TCGA revealed that there was no significant difference in HERC4 mRNA between CRC tissues with and without lymphatic metastasis, ns: no significance. **d** No significant changes were found in HERC4 mRNA expression between CRC tissues with and without distant metastasis in TCGA. ns: no significance.

**Additional file 3: Figure S2.** Representative images of HE staining of lung sections displayed metastatic nodules in sh-NC, sh-circHERC4-1 and sh-circHERC4-2 groups.

**Additional file 4: Figure S3.** Representative images of HE staining of liver sections displayed metastatic nodules in sh-NC, sh-circHERC4-1 and sh-circHERC4-2 groups.

**Additional file 5: Figure S4.** Correlation between circHERC4 and miR-556-5p in 120 CRC tissues.

**Additional file 6: Figure S5.** Relative expression level of CTBP2 mRNA in our 120-patients cohort. **a** We determined the significantly higher expression level of CTBP2 mRNA in CRC tissues compared with paired adjacent normal tissues by qPCR. **b** We detected higher CTBP2 expression in patients had lymphatic metastasis. **c** CTBP2 was associated with distant metastasis. **, *P* < 0.01, ***, *P* < 0.001.

**Additional file 7: Figure S6.** Immunostaining of CTBP2 expression in xenografts tumor tissues after intratumoral circHERC4 silence.

**Additional file 8: Figure S7.** Immunostaining of E-ca expression in xenografts tumor tissues after intratumoral circHERC4 inhibition.

**Additional file 9: Figure S8.** Immunostaining of Ki67 expression in xenografts tumor tissues after intratumoral circHERC4 inhibition.

**Additional file 10: Figure S9.** CircHERC4, miR-556-5p and CTBP2 were examined using FISH and IHC in a 20-patients cohort consist of 10 low-grade and 10 high-grade CRC samples contained in our 120-patients cohort. **a** Represent images of FISH in 20 paired CRC tissues. **b** CircHERC4 was upregulated in high-grade CRC tissues and miR-556-5p was downregulated in low-grade samples. **c** Correlation between circHERC4 and miR-556-5p.

**Additional file 11: Figure S10.** In our in vivo rescue experiment, immunostaining of CTBP2 expression was upregulated when circHERC4 was overexpressed and this effect could be rescued by CTBP2 siRNA and miR-556-5p mimic.

**Additional file 12: Figure S11.** Immunostaining of E-ca expression in xenografts tumor tissues in our rescue experiment. E-ca expression level was upregulated in “circHERC4 OE” group and was rescued by CTBP2 siRNA and miR-556-5p mimic.

**Additional file 13: Figure S12.** Immunostaining of Ki67 expression in xenografts tumor tissues in our rescue experiment. The promotional effect of circHERC4 on Ki67 could be rescued by CTBP2 siRNA and miR-556-5p mimic.

**Additional file 14: Figure S13.** Representative images of HE staining of lung sections displayed metastatic nodules from in vivo rescue experiment.

**Additional file 15: Figure S14.** Representative images of HE staining of liver sections displayed metastatic nodules from in vivo rescue experiment.

## Data Availability

The datasets used and/or analyzed during the current study are available from the corresponding author upon reasonable request.

## Abbreviations

CRC: Colorectal cancer
CCK8: Cell counting kit-8
circHERC4: Hsa_circ_0007113
RNA-seq: RNA sequencing
IHC: Immunohistochemistry
qRT-PCR: Quantitative real-time PCR
siRNA: Short interfering RNA
shRNA: Short hairpin RNA
CTBP2: C-terminal-binding protein 2
E-ca: E-cadherin
