# Supplementary material for: Circular RNA circHERC4 as a novel oncogenic driver to promote tumor metastasis via the miR-556-5p/CTBP2/E-cadherin axis in colorectal cancer
Source: J Hematol Oncol. 2021 Nov 15;14:194. doi: 10.1186/s13045-021-01210-2 (PMC8591961; doi:10.1186/s13045-021-01210-2)
Supplement: Supplementary file 1 — Additional file 1: Table S1. Primer sequences for the qRT-PCR. Table S2. microRNA primers for the qRT-PCR. Table S3. siRNA sequence. Table S4. DNA oligo sequences for pull-down assays. Table S5. Sequences of the FISH probes. [file 13045_2021_1210_MOESM1_ESM.docx]

**Supplementary tables**

**Table S1：**Primers for Real-time PCR

| Gene | Oligo name | Oligo sequence |
| --- | --- | --- |
| HERC4  circHERC4  Malat 1  MT COI  GAPDH  U6 | Forward primer  Reverse primer  Forward primer  Reverse primer  Forward primer  Reverse primer  Forward primer  Reverse primer  Forward primer  Reverse primer  Forward primer  Reverse primer | CACACTTCTGCTTTTGTTCCTTC  GGCTTTTCCTGTTGCTTGTTG  CTTGTGGACGAGCAGGTTGTT  CTGATCCTACCAGGCCAAGCT  GTGATGCGAGTTGTTCTCCG  CTGGCTGCCTCAATGCCTAC  GACGTAGACACACGAGCATATTTCA  AGGACATAGTGGAAGTGAGCTACAAC  ATCACCATCTTCCAGGAGCGA  CCTTCTCCATGGTGGTGAAGAC  CTCGCTTCGGCAGCACA  AACGCTTCACGAATTTGCGT |

**Table S2：**MicroRNA primers

| Gene | Oligo sequence |
| --- | --- |
| miR-556-5p  miR-15a-5p  miR-15b-5p  miR-16-5p  miR-338-5p  miR-502-5p  miR-921 | GATGAGCTCATTGTAAT  TAGCAGCACATAATGGT  TAGCAGCACATCATGGT  TAGCAGCACGTAAATAT  AACAATATCCTGGTGCT  ATCCTTGCTATCTGGGT  ACTAGTGACGGACAGA |

**Table S3：**siRNA sequence

| Name | Oligo sequence |
| --- | --- |
| circHERC4 siRNA1  circHERC4 siRNA2  CTBP2 siRNA | TTGCTTGTGGACGAGCAGGTT  TTGTGGACGAGCAGGTTGTTG  GUGAUCGUGCGGAUAGGCAGU |

**Table S4：**RNA pulldown probe sequence

| Name | Oligo sequence |
| --- | --- |
| circHERC4-5＇bio  HERC4-5＇bio-1  HERC4-5＇bio-2  HERC4-5＇bio-3 | ACAACCTGCTCGTCCACAAG  TAACGTATGAGCTTCTCCAC  TGCTTTTTACAGTCAGTACC  CAGTATGATCTTCTCCACAA |

**Table S5:** FISH sequence

| Name | Oligo sequence |
| --- | --- |
| circHERC4 (FISH)  miR-556-5p(FISH) | GGGCAACAACCTGCTCGTCCACAAGCAATC  CTCATATTACAATGAGCTCATC |
