# Supplementary figures and images for: Circular RNA circHERC4 as a novel oncogenic driver to promote tumor metastasis via the miR-556-5p/CTBP2/E-cadherin axis in colorectal cancer
Source: J Hematol Oncol. 2021 Nov 15;14:194. doi: 10.1186/s13045-021-01210-2 (PMC8591961; doi:10.1186/s13045-021-01210-2)

**A**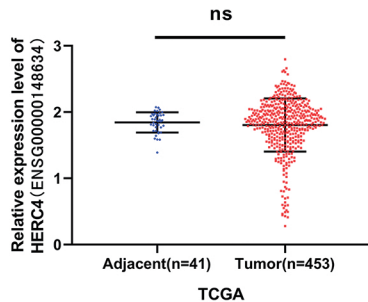**B**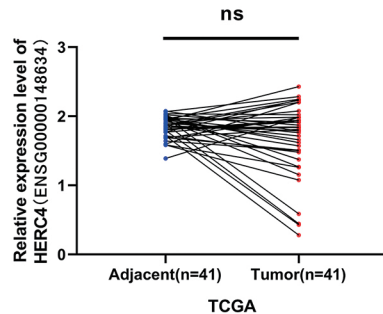**C**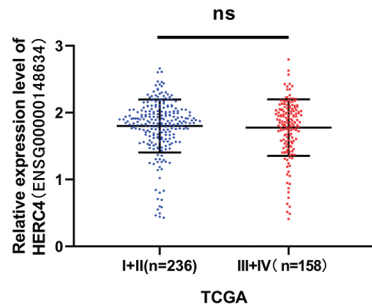**D**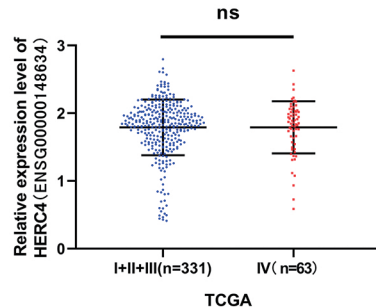

Supplement: Supplementary file 2 — Additional file 2: Figure S1. Clinical significance of HERC4 mRNA in TCGA. a Relative expression level of HERC4 mRNA in CRC tissues and paired adjacent normal tissues derived from TCGA, ns: no significance. b HERC4 mRNA level in paired tissues in TCGA. c Data from TCGA revealed that there was no significant difference in HERC4 mRNA between CRC tissues with and without lymphatic metastasis, ns: no significance. d No significant changes were found in HERC4 mRNA expression between CRC tissues with and without distant metastasis in TCGA. ns: no significance. [file 13045_2021_1210_MOESM2_ESM.pdf]

Lung

sh-NC

#1

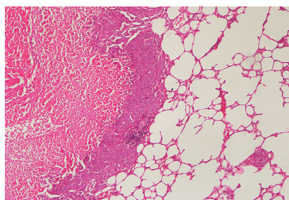

#2

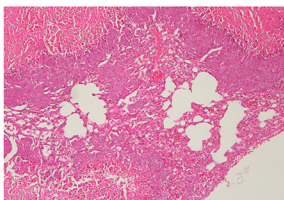

#3

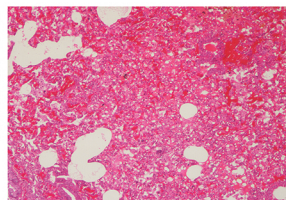

#4

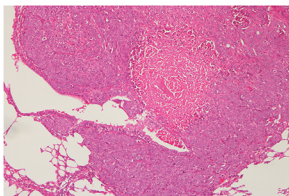

#5

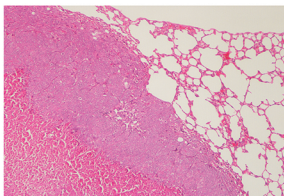

#6

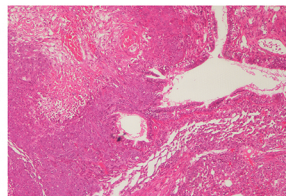

sh-circHERC4-1

#1

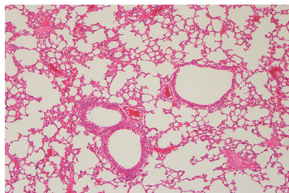

#2

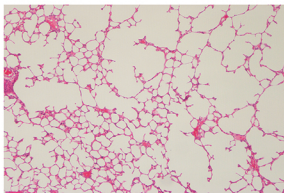

#3

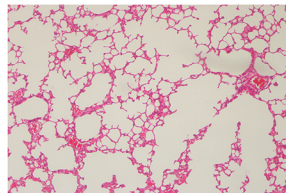

#4

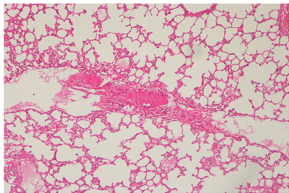

#5

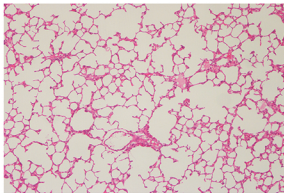

#6

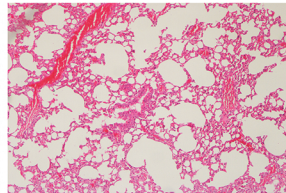

sh-circHERC4-2

#1

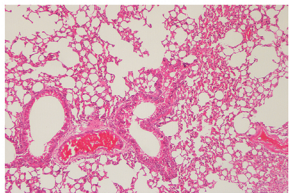

#2

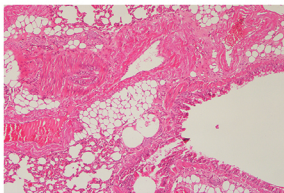

#3

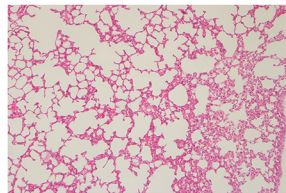

#4

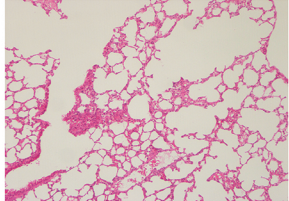

#5

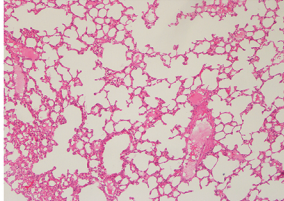

#6

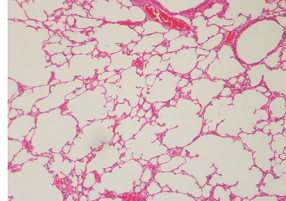

Supplement: Supplementary file 3 — Additional file 3: Figure S2. Representative images of HE staining of lung sections displayed metastatic nodules in sh-NC, sh-circHERC4-1 and sh-circHERC4-2 groups. [file 13045_2021_1210_MOESM3_ESM.pdf]

## Liver

sh-NC

## #1

## #2

### #3

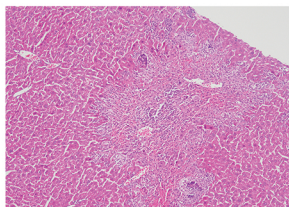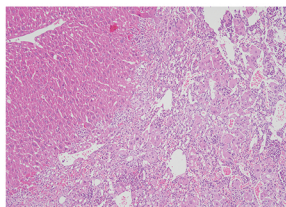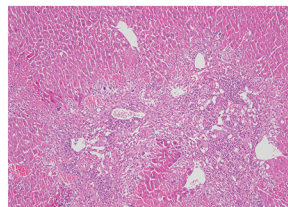

#### #4

## #5

## #6

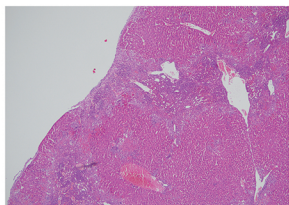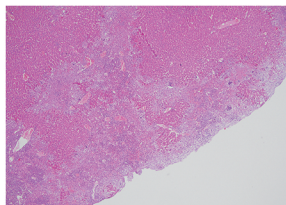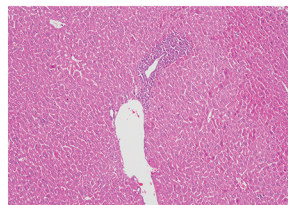

## #1

## #2

### #3

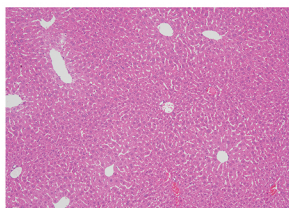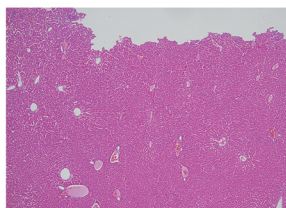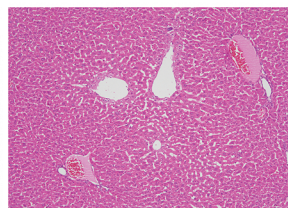

#### #4

## #5

## #6

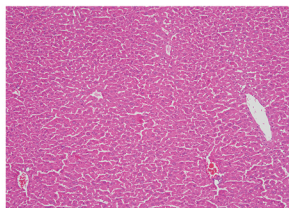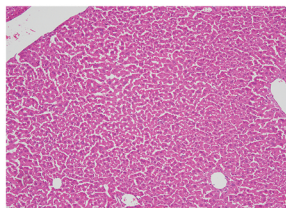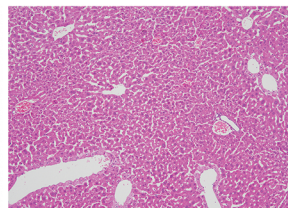

## #1

## #2

### #3

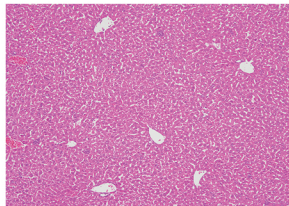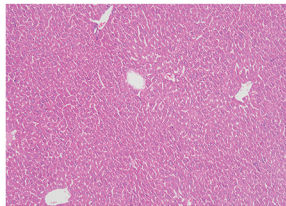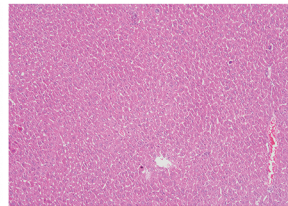

#### #4

## #5

## #6

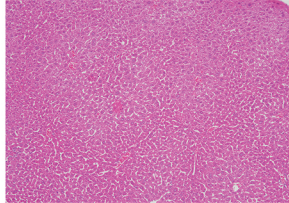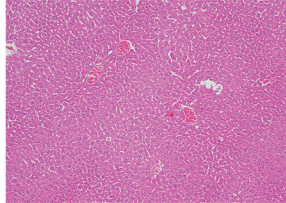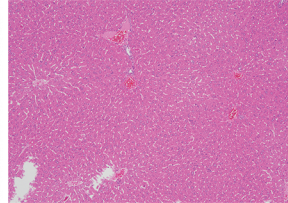

## sh-circHERC4-2

Supplement: Supplementary file 4 — Additional file 4: Figure S3. Representative images of HE staining of liver sections displayed metastatic nodules in sh-NC, sh-circHERC4-1 and sh-circHERC4-2 groups. [file 13045_2021_1210_MOESM4_ESM.pdf]

$P=0.034$   $t=-0.19$

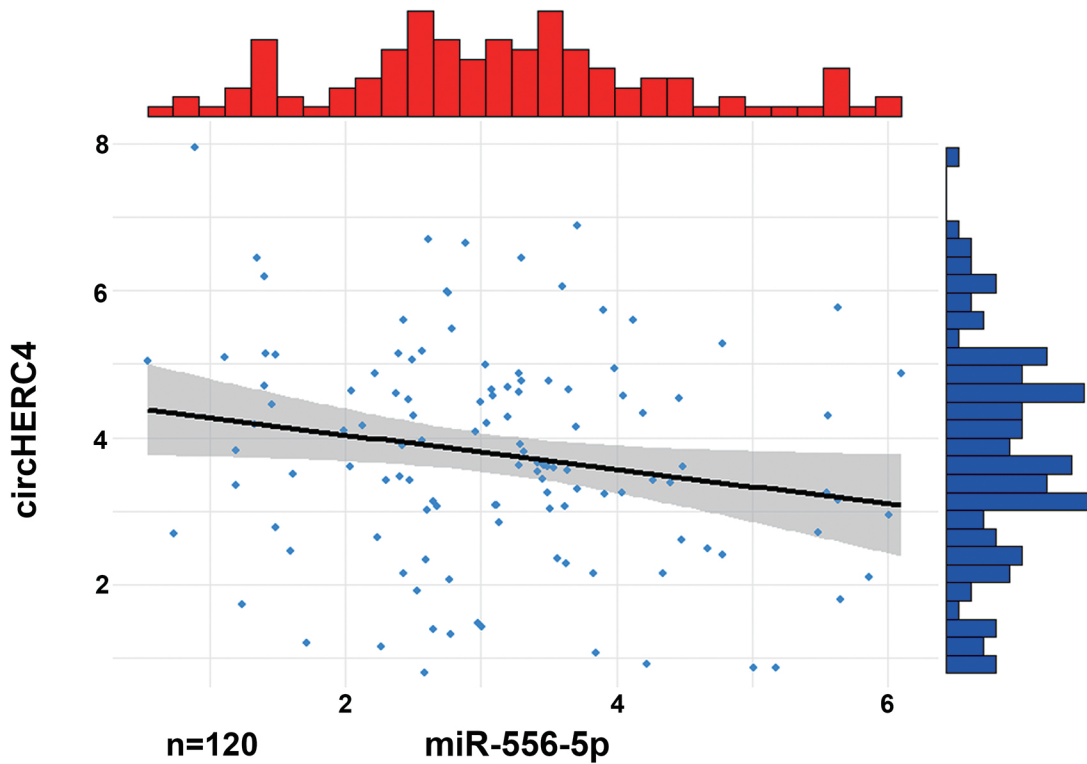

Supplement: Supplementary file 5 — Additional file 5: Figure S4. Correlation between circHERC4 and miR-556-5p in 120 CRC tissues. [file 13045_2021_1210_MOESM5_ESM.pdf]

**A**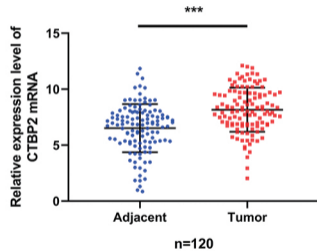**B**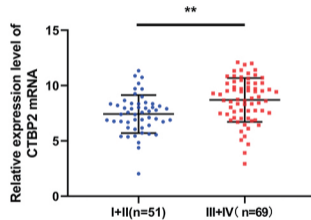**C**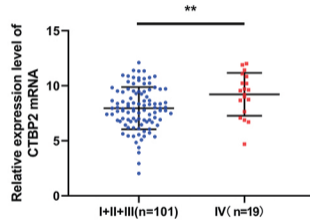

Supplement: Supplementary file 6 — Additional file 6: Figure S5. Relative expression level of CTBP2 mRNA in our 120-patients cohort. a We determined the significantly higher expression level of CTBP2 mRNA in CRC tissues compared with paired adjacent normal tissues by qPCR. b We detected higher CTBP2 expression in patients had lymphatic metastasis. c CTBP2 was associated with distant metastasis. **, P < 0.01, ***, P < 0.001. [file 13045_2021_1210_MOESM6_ESM.pdf]

CTBP2

sh-NC

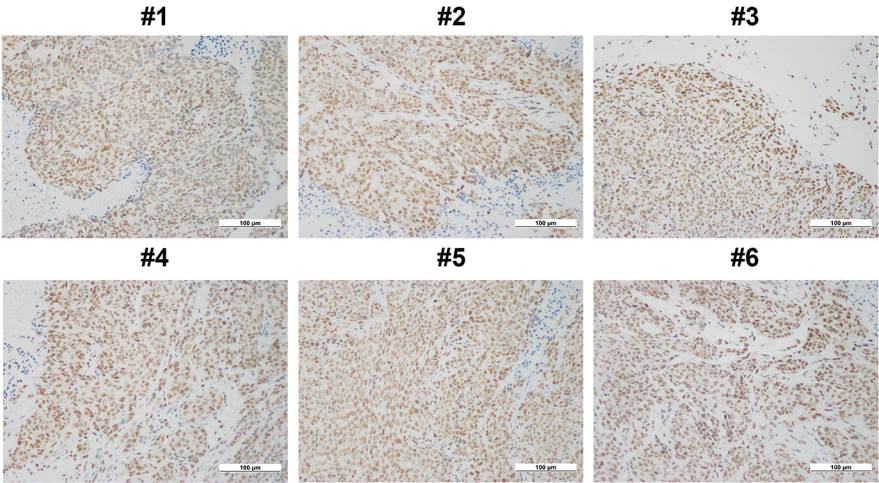

sh-circHERC4-1

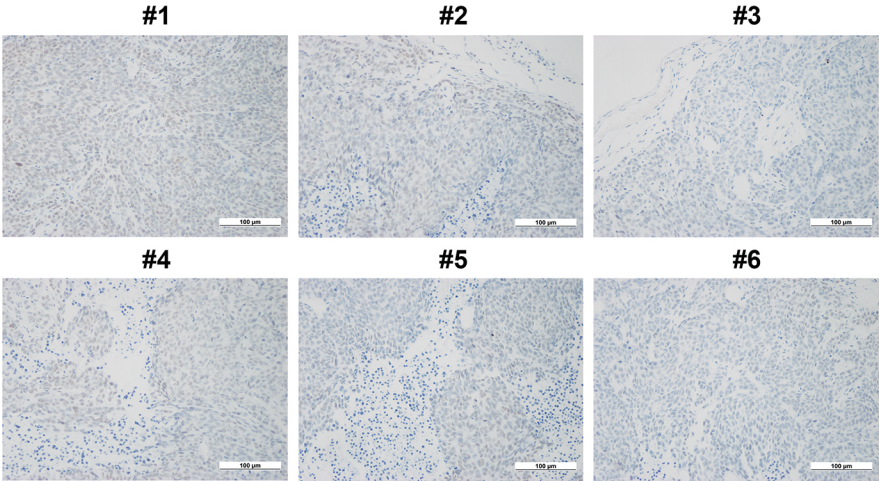

sh-circHERC4-2

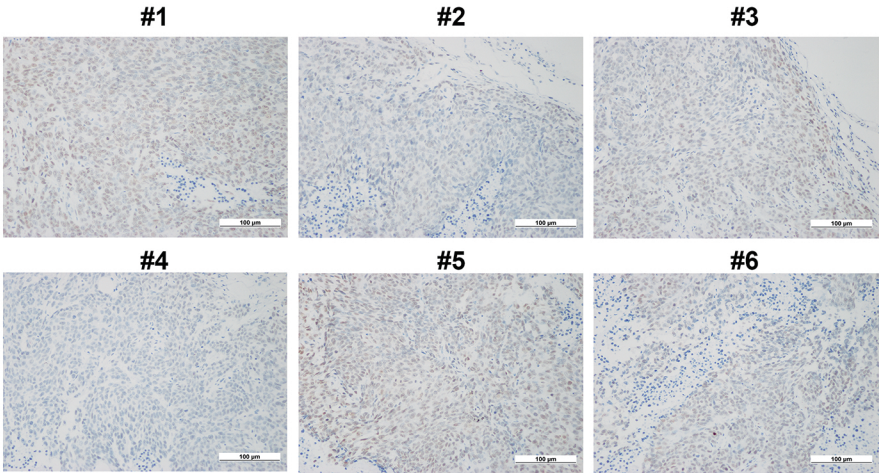

Supplement: Supplementary file 7 — Additional file 7: Figure S6. Immunostaining of CTBP2 expression in xenografts tumor tissues after intratumoral circHERC4 silence. [file 13045_2021_1210_MOESM7_ESM.pdf]

E-ca

sh-NC

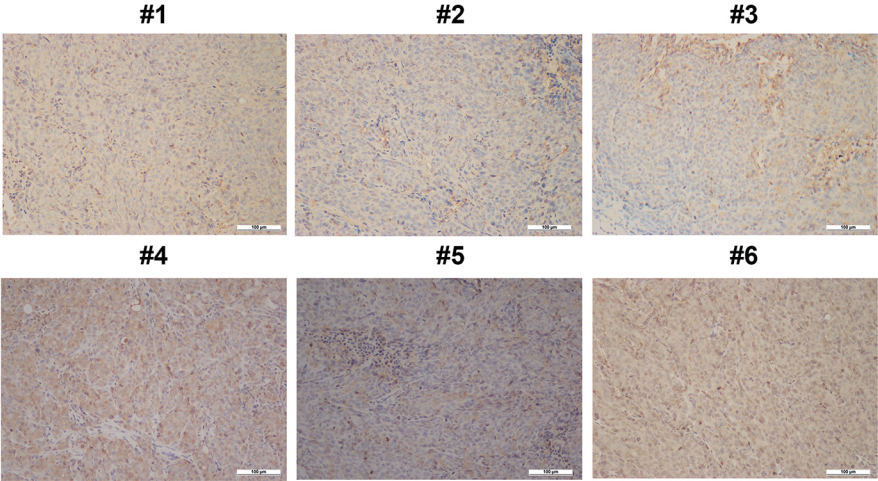

sh-circHERC4-1

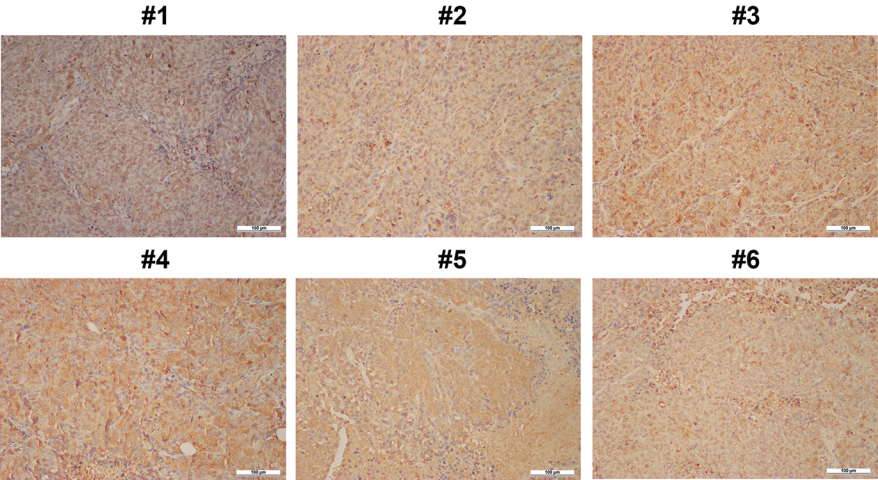

sh-circHERC4-2

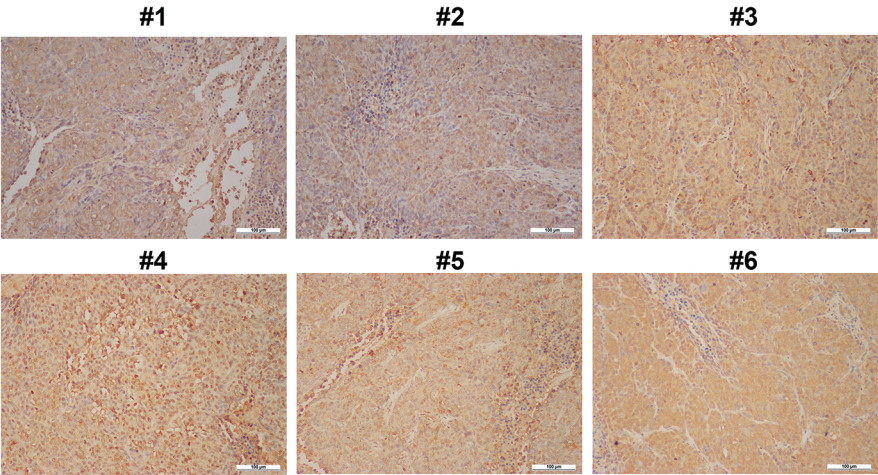

Supplement: Supplementary file 8 — Additional file 8: Figure S7. Immunostaining of E-ca expression in xenografts tumor tissues after intratumoral circHERC4 inhibition. [file 13045_2021_1210_MOESM8_ESM.pdf]

**Ki67**

**sh-NC**

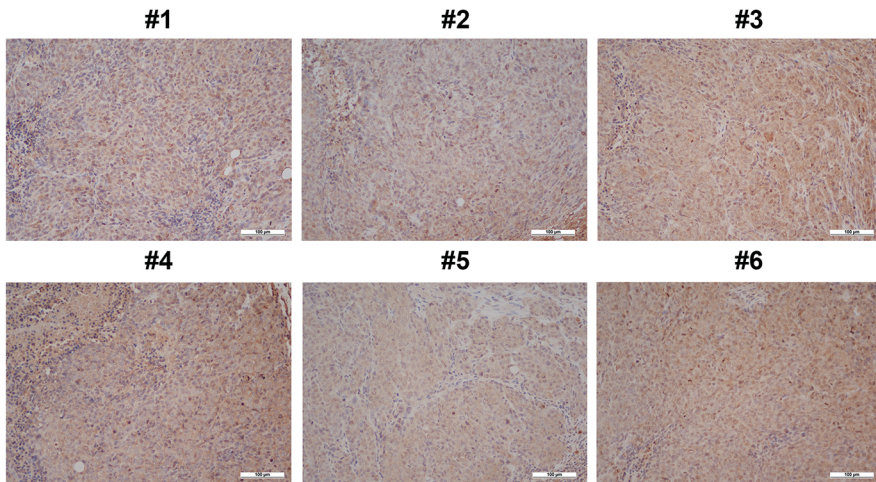

**sh-circHERC4-1**

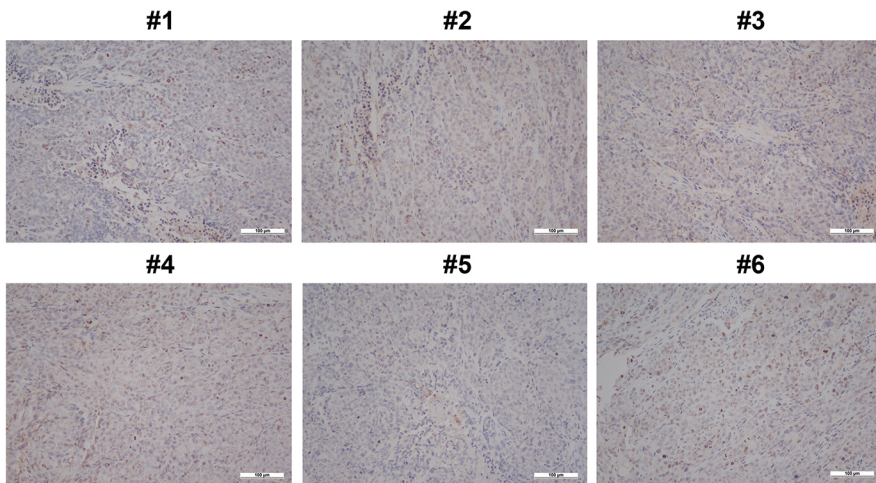

**sh-circHERC4-2**

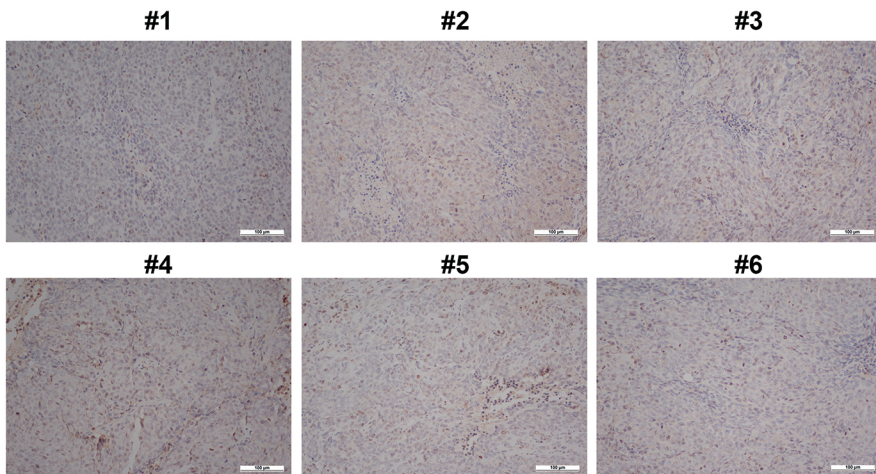

Supplement: Supplementary file 9 — Additional file 9: Figure S8. Immunostaining of Ki67 expression in xenografts tumor tissues after intratumoral circHERC4 inhibition. [file 13045_2021_1210_MOESM9_ESM.pdf]

**A**

High-grade

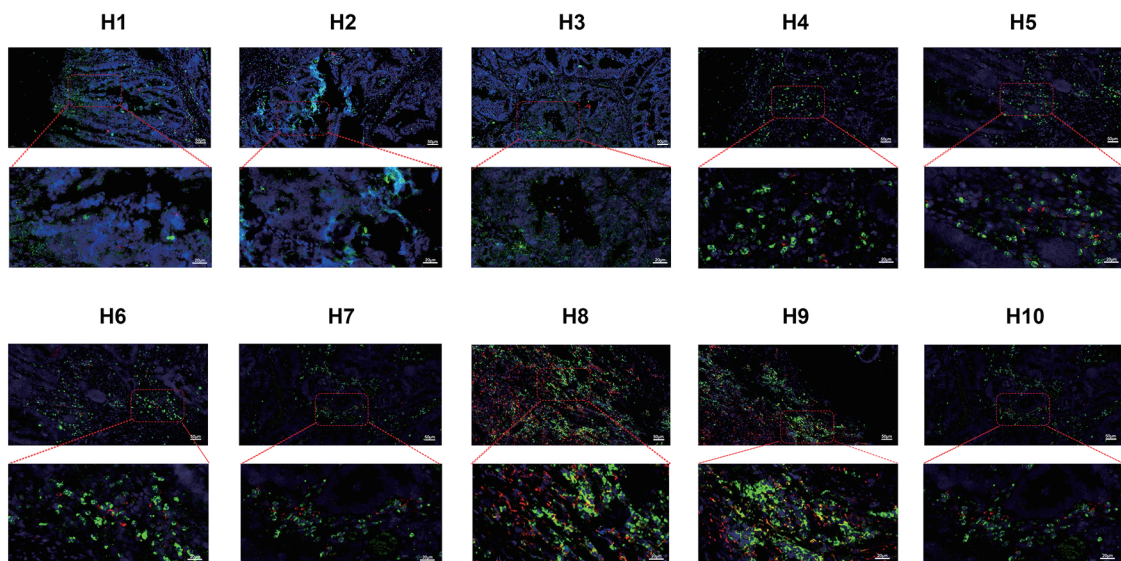

Low-grade

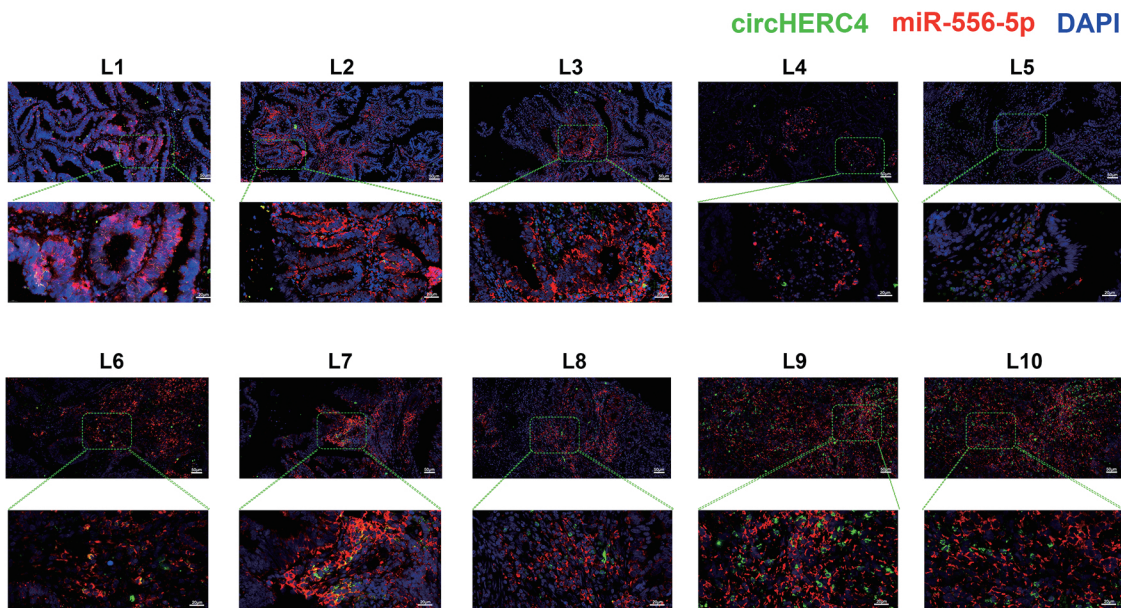**B**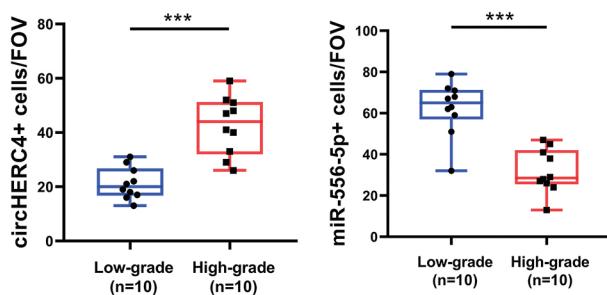**C**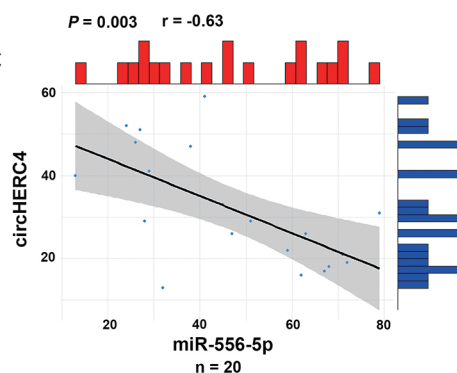

Supplement: Supplementary file 10 — Additional file 10: Figure S9. CircHERC4, miR-556-5p and CTBP2 were examined using FISH and IHC in a 20-patients cohort consist of 10 low-grade and 10 high-grade CRC samples contained in our 120-patients cohort. a Represent images of FISH in 20 paired CRC tissues. b CircHERC4 was upregulated in high-grade CRC tissues and miR-556-5p was downregulated in low-grade samples. c Correlation between circHERC4 and miR-556-5p. [file 13045_2021_1210_MOESM10_ESM.pdf]

CTBP2

Vector

circHERC4 OE+CTBP2 siRNA

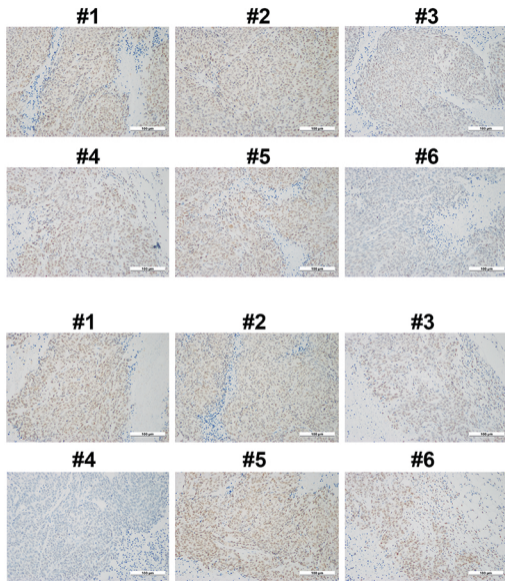

CTBP2

circHERC4 OE

circHERC4 OE+miR-556-5p mimic

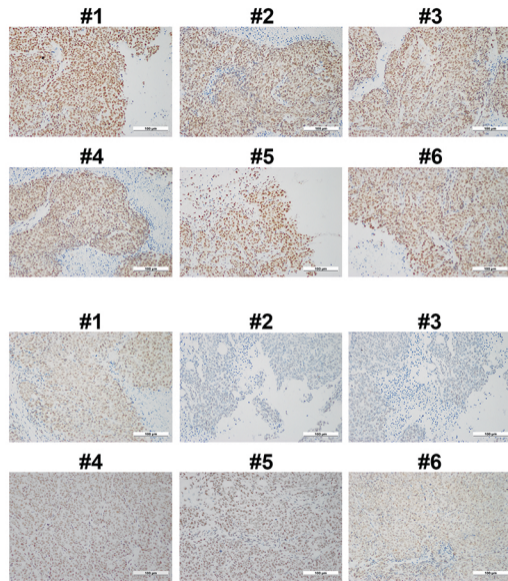

Supplement: Supplementary file 11 — Additional file 11: Figure S10. In our in vivo rescue experiment, immunostaining of CTBP2 expression was upregulated when circHERC4 was overexpressed and this effect could be rescued by CTBP2 siRNA and miR-556-5p mimic. [file 13045_2021_1210_MOESM11_ESM.pdf]

E-ca

Vector

circHERC4 OE+CTBP2 siRNA

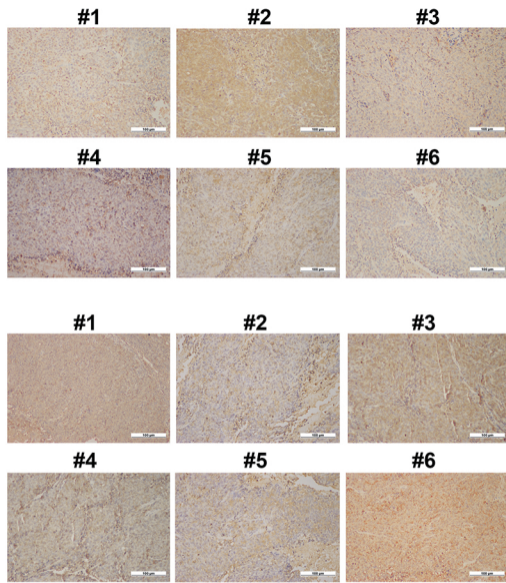

E-ca

circHERC4 OE

circHERC4 OE+miR-556-5p mimic

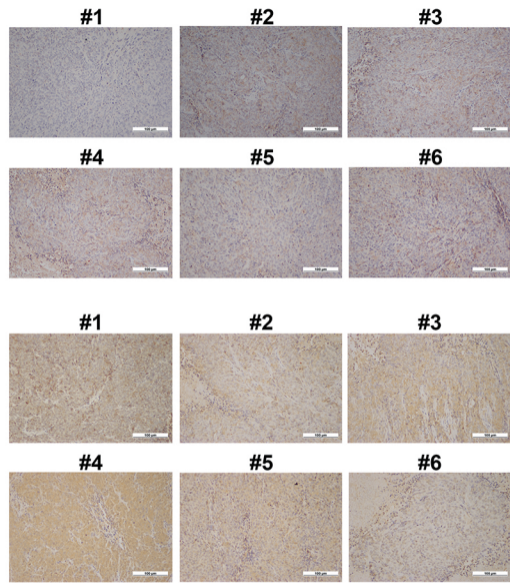

Supplement: Supplementary file 12 — Additional file 12: Figure S11. Immunostaining of E-ca expression in xenografts tumor tissues in our rescue experiment. E-ca expression level was upregulated in “circHERC4 OE” group and was rescued by CTBP2 siRNA and miR-556-5p mimic. [file 13045_2021_1210_MOESM12_ESM.pdf]

Ki67

Vector

circHERC4 OE+CTBP2 siRNA

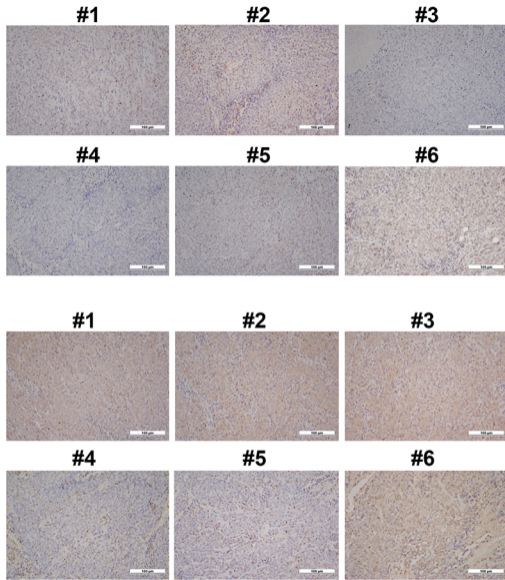

Ki67

circHERC4 OE

circHERC4 OE+miR-556-5p mimic

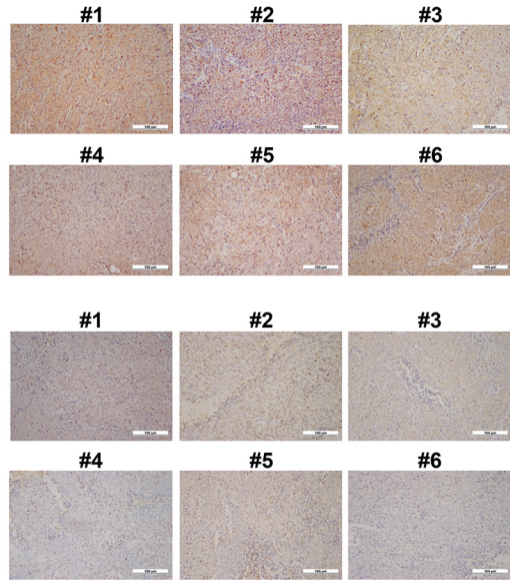

Supplement: Supplementary file 13 — Additional file 13: Figure S12. Immunostaining of Ki67 expression in xenografts tumor tissues in our rescue experiment. The promotional effect of circHERC4 on Ki67 could be rescued by CTBP2 siRNA and miR-556-5p mimic. [file 13045_2021_1210_MOESM13_ESM.pdf]

Lung

circHERC4 OE+CTBP2 siRNA

Vector

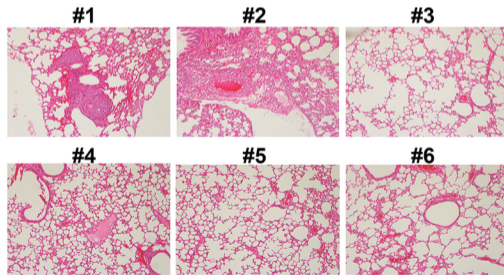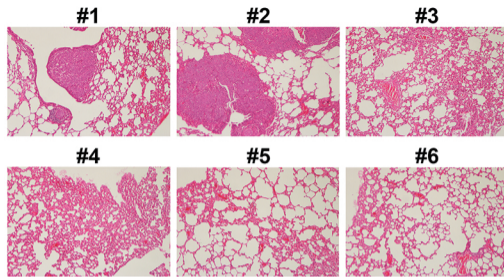

Lung

circHERC4 OE+miR-556-5p mimic

circHERC4 OE

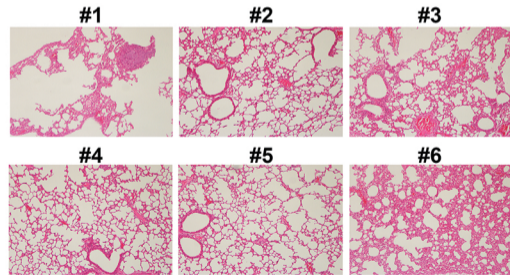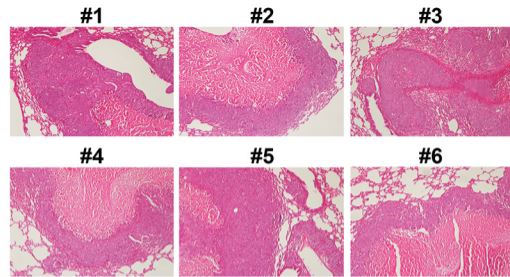

Supplement: Supplementary file 14 — Additional file 14: Figure S13. Representative images of HE staining of lung sections displayed metastatic nodules from in vivo rescue experiment. [file 13045_2021_1210_MOESM14_ESM.pdf]

Liver

circHERC4 OE+CTBP2 siRNA

Vector

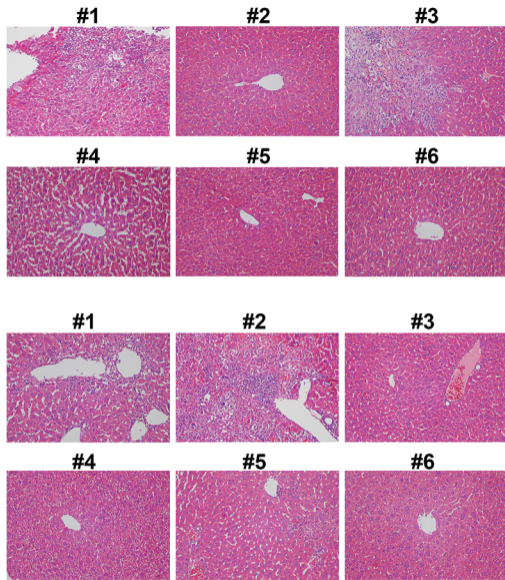

Liver

circHERC4 OE+miR-556-5p mimic

circHERC4 OE

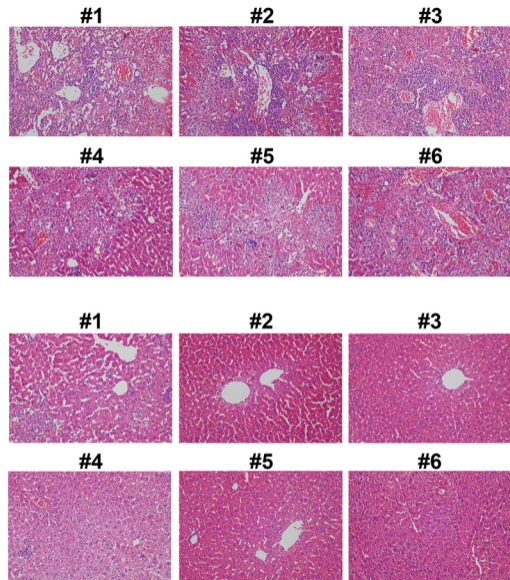

Supplement: Supplementary file 15 — Additional file 15: Figure S14. Representative images of HE staining of liver sections displayed metastatic nodules from in vivo rescue experiment. [file 13045_2021_1210_MOESM15_ESM.pdf]
